# CHMP2A regulates tumor sensitivity to natural killer cell-mediated cytotoxicity

**DOI:** 10.1038/s41467-022-29469-0

**Published:** 2022-04-07

**Authors:** Davide Bernareggi, Qi Xie, Briana C. Prager, Jiyoung Yun, Luisjesus S. Cruz, Timothy V. Pham, William Kim, Xiqing Lee, Michael Coffey, Cristina Zalfa, Pardis Azmoon, Huang Zhu, Pablo Tamayo, Jeremy N. Rich, Dan S. Kaufman

**Affiliations:** 1 Department of Medicine, University of California San Diego, La Jolla, CA USA; 2 Key Laboratory of Growth Regulation and Translational Research of Zhejiang Province, School of Life Sciences, Westlake University, Hangzhou, Zhejiang China; 3 Westlake Laboratory of Life Sciences and Biomedicine, Hangzhou, Zhejiang China; 4 Institute of Basic Medical Sciences, Westlake Institute for Advanced Study, Hangzhou, Zhejiang Province China; 5 Cleveland Clinic Lerner College of Medicine at Cleveland Clinic & Case Western Reserve University, Cleveland, OH USA; 6 Center for Novel Therapeutics and Moores Cancer Center, UCSD, San Diego, CA USA; 7 Division of Medical Genetics, Department of Medicine, Moores Cancer Center, University of California San Diego, La Jolla, CA USA; 8 Department of Oncology, Henan Provincial People’s Hospital, Zhengzhou University People’s Hospital, Zhengzhou, Henan China; 9 Department of Immunology and Microbiology, The Scripps Research Institute, La Jolla, CA USA; 10 Department of Pathology, University of California San Diego, La Jolla, CA USA

**Keywords:** Tumour immunology, CNS cancer, Head and neck cancer, Innate lymphoid cells

## Abstract

Natural killer (NK) cells are known to mediate killing of various cancer types, but tumor cells can develop resistance mechanisms to escape NK cell-mediated killing. Here, we use a “two cell type” whole genome CRISPR-Cas9 screening system to discover key regulators of tumor sensitivity and resistance to NK cell-mediated cytotoxicity in human glioblastoma stem cells (GSC). We identify CHMP2A as a regulator of GSC resistance to NK cell-mediated cytotoxicity and we confirm these findings in a head and neck squamous cells carcinoma (HNSCC) model. We show that deletion of CHMP2A activates NF-κB in tumor cells to mediate increased chemokine secretion that promotes NK cell migration towards tumor cells. In the HNSCC model we demonstrate that CHMP2A mediates tumor resistance to NK cells via secretion of extracellular vesicles (EVs) that express MICA/B and TRAIL. These secreted ligands induce apoptosis of NK cells to inhibit their antitumor activity. To confirm these in vitro studies, we demonstrate that deletion of CHMP2A in CAL27 HNSCC cells leads to increased NK cell-mediated killing in a xenograft immunodeficient mouse model. These findings illustrate a mechanism of tumor immune escape through EVs secretion and identify inhibition of CHMP2A and related targets as opportunities to improve NK cell-mediated immunotherapy.

## Abstract (web summary)

Genetic alteration can render tumor cells resistant to immune cell-mediated killing. Here based on a genome-wide CRISPR screening, the authors show that expression of CHMP2A confers tumor cell resistance to NK cell-mediated cytotoxicity, mechanistically involving CHMP2A-dependent regulation of extracellular vesicle secretion.

## Introduction

Natural killer (NK) cells play a key role in tumor-immune surveillance owing to their ability to identify and kill both hematological malignancies and solid tumors, including control of metastatic disease^1–3^. Genomic studies demonstrated multiple and diverse genetic alterations in cancer^4^ with some mutations that can induce resistance^5^ or increase sensitivity to immune cells^6^. Although many NK cell-activating and inhibitory ligands have been identified^3^, these do not fully explain the mechanisms that may make tumor cells sensitive or resistant to NK cell-mediated activity. Recently, “two cell type” (TCT) CRISPR-Cas9 screens have been used to mimic the mutational multiplicity of tumors to identify novel mechanisms that regulate NK cell or T-cell-mediated antitumor activity^7–10^.

Glioblastoma multiforme (GBM) is a heterogeneous brain tumor with a complex mutational pattern and an intricate tumor microenvironment (TME)^11^. Intratumor heterogeneity in GBM is sustained by glioblastoma stem cells (GSCs) that drive resistance to therapy^11^. Therefore, GSCs provide an important cell population to identify mechanisms of resistance and previously unidentified targets for NK cell-based immunotherapy. Here, we utilized a TCT-screening approach to identify key genes that increased or reduced the sensitivity of GSC to NK cell-mediated cytotoxicity. Since head and neck squamous cells carcinoma (HNSCC) is among the most highly immune-infiltrated cancer types with a high degree of infiltration of NK cells, which correlates significantly with patient’s survival^12,13^, we subsequently validated these findings on this model.

Extracellular vesicles (EVs) are emerging as a key component in the biogenesis of GBM and HNSCC, promoting migration, invasion, persistence, and contribute to the modification of the TME to support tumor progression^14–19^. Indeed, EVs have been proposed as biomarker for the diagnosis of GBM^20–22^ and more recently Hoshino and colleagues^23^ have proposed to use EVs as biomarkers to identify multiple types of cancers. Ligands for NK cell-activating receptors are often upregulated on tumor cells or during infection^24^, and the loss of these ligands reduces NK cell recognition and killing. MHC Class I Polypeptide-Related Sequence A/B (MICA/B) are ligands expressed on tumor cells for the NK-activating receptor NK group 2 member D (NKG2D)^24^; however, some tumors including leukemia, prostate cancer, melanoma, breast, lung, ovarian, and colon carcinomas frequently downregulate or shed these ligands to limit the cytotoxicity of NK cells^5,25–27^. NKG2D ligands can be secreted on the surface of EVs, impairing NK cells’ functions^26–30^. Indeed, EVs-bearing MICA/B can act as decoys and inhibit NK cells^26^. Therefore, blocking the shedding of NKG2D ligands can increase NK cells’ antitumor activity^31^. Tumor-derived EVs carrying tumor necrosis factor-related apoptosis-inducing ligand (TRAIL) and Fas ligand (FasL) can induce apoptosis in NK cells and CD8^+^ T lymphocytes^32–35^.

Chemokines can promote the recruitment of NK cells to the TME to regulate NK cell-mediated antitumor activity^36^. CXCL10 increases NK cell migration^37^, and secretion of CXCL10 has been correlated with reduced tumor growth in xenograft models of lymphoma, squamous cell carcinoma, and adenocarcinoma of the lung^38^. In a model of GBM-expressing CXCL12, Muller et al.^39^ modified epidermal growth factor receptor (EGFR)-III expressing chimeric antigen receptor (CAR) NK cells to overexpress CXCR4, showing improved migration and a decrease in tumor volume.

Here, we identify chromatin-modifying protein/charged multivesicular body protein (CHMP2A) as a target for increasing GSC and HNSCC cell sensitivity to NK cell-mediated killing. CHMP2A is a subunit of the endosomal sorting complexes required for transport III (ESCRT-III), a molecular complex involved in the formation of multivesicular bodies^40^ and EVs biogenesis^41,42^. We demonstrate the loss of *CHMP2A* increases GSC and HNSCC cell sensitivity to NK cell-mediated killing in vitro, as well as in vivo using an HNSCC xenograft model. *CHMP2A*-knockout (KO) tumor cells secrete more CXCL10 and CXCL12, increasing NK cell migration in vitro. Moreover, since we observe that tumor-derived EVs can inhibit NK cell activity through MICA/B and TRAIL, deletion of *CHMP2A* limits the secretion of EVs by the tumor cells leading to enhanced NK cell-mediated cytotoxicity. Together, these studies demonstrate that CHMP2A-mediated activity provides a target whose ablation or inactivation can increase NK cell-mediated antitumor activity.

## Results

### Genome-wide CRISPR screening of GSC identifies key genes regulating NK cell activity

**Fig. 1:**
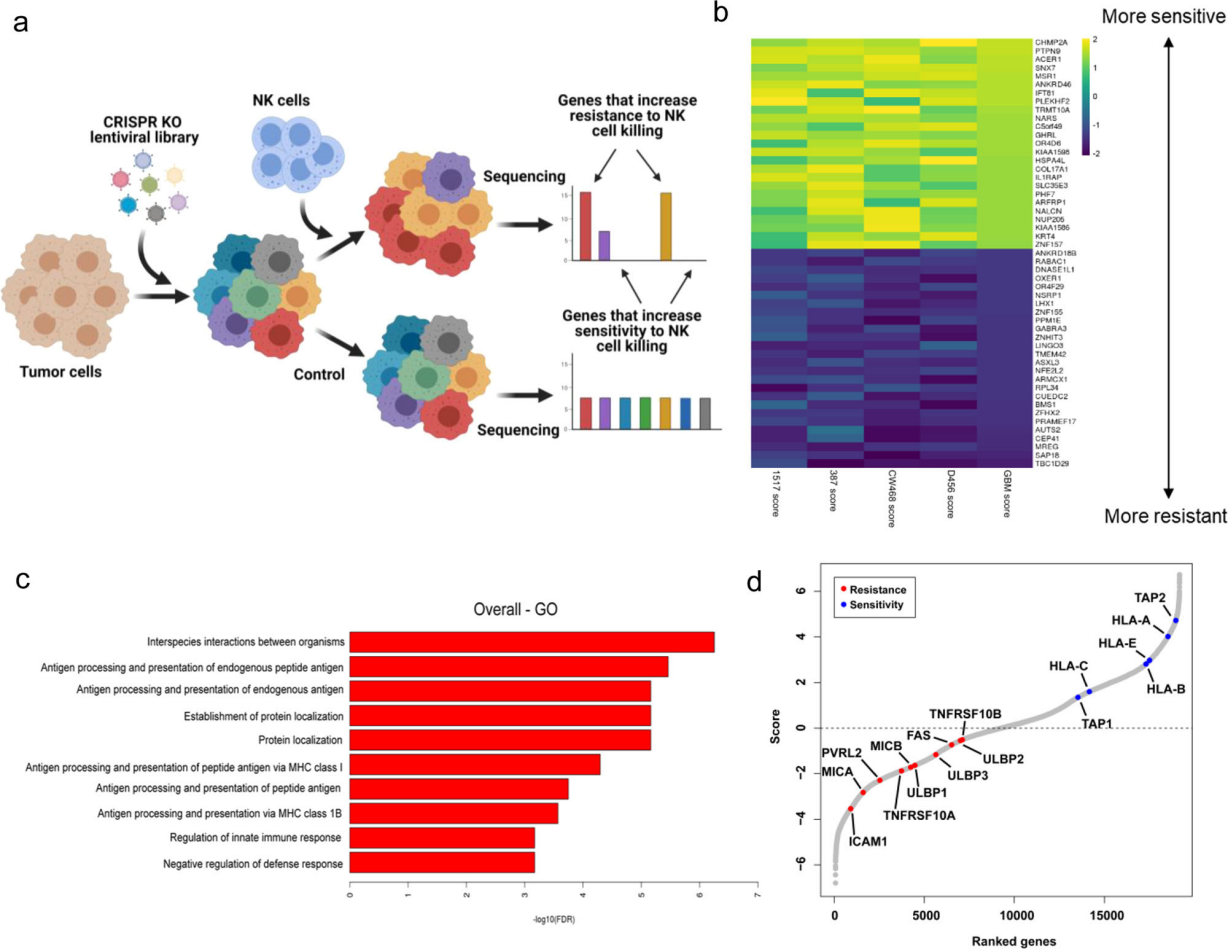
Two cell-type CRISPR/Cas9 screen using NK cell-mediated killing of GSCs. **a** Schematic of screen design. Screening hits in GSC/NK cell co-culture (*n* = 2 samples per cell line) were compared with GSC monoculture to identify genes that induce resistance or sensitivity to NK cell killing (created with Biorender.com). **b** Heatmap of individual and averaged scores of top 25 genes that increase sensitivity or resistance across four GSC models. **c** GO gene set enrichment analysis of hits. *Y* axis indicates −log10 FDR of gene set enrichment. **d** Scatter plot showing the stratification of genes identified by the GSC CRISPR/Cas9 two cell-type screen ordered by score. Highlighted in red are known genes known that typically activate NK cells (MICA, MICB, ULBP1, ULBP2, ULBP3, FAS, TNFRSF10A [TRAIL-R1], TNFRSF10B [TRAIL-R2], and ICAM1; and highlighted in blue are genes are known to inhibit NK cell-mediated activity (HLA-A, HLA-B, HLA-C, HLA-E, TAP1, and TAP2).

We performed a TCT genome-wide CRISPR screen in four GSC lines to identify perturbations that modulate NK cells-mediated killing activity (Fig. 1a). GSC 1517, 387, CW468, and D456 were lentivirally transduced to express the Brunello short-guide RNA (sgRNA) library and Cas9 endonuclease. Nine days post infection, cells were either cultured without effectors or challenged with peripheral blood NK cells at a 2:1 E:T ratio for 24-h. We compared changes in sgRNA abundance between the challenged and unchallenged GSC ranking the genes using MAGeCK^43^ and the top 25 hits that increased sensitivity or resistance to NK cells killing were ranked by RIGER using the Kolmogorov–Smirnov algorithm (Fig. 1b). The screen revealed that in NK cell-sensitive GSC, target genes were enriched in the ER-phagosome pathway, antigen presentation, cellular localization, and regulation of innate immune response (Fig. 1c). Cells lacking the proteins encoded from the major histocompatibility gene complex (HLA) are a natural target of NK cells and, as expected, the KO of genes involved in antigen presentation like HLA-A, HLA-B, HLA-C, HLA- E, as well as for TAP1 and TAP2 increased GSC sensitivity to NK cells (Fig. 1d). We observed an expected stratification of some NK cell-activating ligands (MICA, MICB, ULBP1, ULBP2, ULBP3, FAS, TRAIL-R1, TRAIL-R2, and ICAM1) that increased GSC resistance once targeted with sgRNAs (Fig. 1d). The whole-genome CRISPR screen revealed CHMP2A as top hit that increased the sensitivity of GSC to NK cell-mediated cytotoxicity (Fig. 1b). CHMP2A is a subunit of the ESCRT-III protein machinery and involved in EVs secretion^41,42^, a pathway used by tumor cells to impair NK cells function^26–29^ and therefore a potential target to improve NK cell-mediated antitumor activity, which we wanted to explore more thoroughly.

### *CHMP2A* KO increased the sensitivity of GSC and HNSCC cells to NK cells-mediated killing

**Fig. 2:**
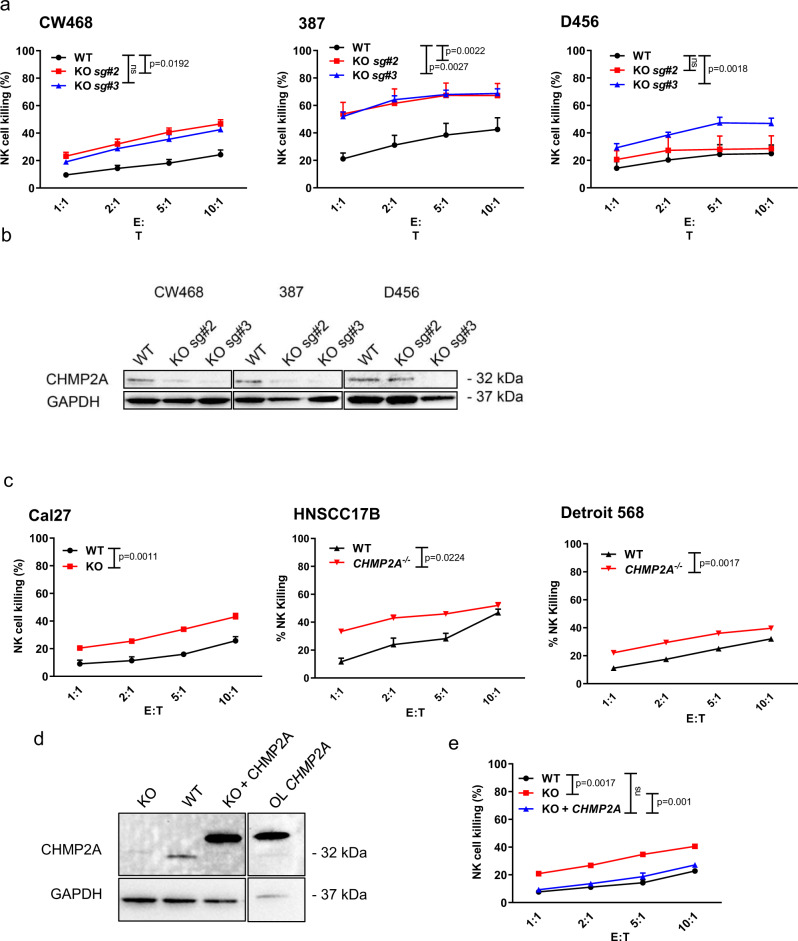
*CHMP2A-*KO increases GSC and HNSCC cell lines sensitivity to NK cell-mediated cytotoxicity. **a** 4 hour cytotoxicity assay to determine the effect of CHMP2A in GSC resistance to NK cell-mediated cytotoxicity. Two independent sgRNAs were used (sg#2, sg#3). **b** immunoblot analysis of WT and KO GSC lines KO for CHMP2A using two sgRNAs (sg#2, sg#3). GAPDH serves as a loading control. **c** 4 hour cytotoxicity assay to determine the effect of CHMP2A in HNSCC-resistance to NK cell-mediated cytotoxicity. **d** Western blot analysis showing the rescue of KO phenotype. Here we show Cal27 cells expressing WT levels of *CHMP2A*, no *CHMP2A* (KO), or complemented *CHMP2A* (KO+ overexpressed CHMP2A). HEK293 *CHMP2A o*verexpression lysate (OL) was used as a control. The overexpressed CHMP2A is fused with DDK and MYC tags explaining the 4 kDa increase in molecular weight. For this western blot, GAPDH was used as a loading control. **e** 4 hour cytotoxicity assay to determine the effect of *CHMP2A* in Cal27 cells expressing WT levels of *CHMP2A*, no *CHMP2A* (KO), or complemented *CHMP2A* (KO+ overexpressed *CHMP2A*). **a**, **c**, **e** The error bars represent standard error from the mean (±SEM) across *n* = 3 replicates. Statistical analysis was performed by one-tailed paired Student *t* test. Representative of *n* = 3 independent experiments.

In our initial studies following the TCT-screening results, we deleted *CHMP2A* using CRISPR-Cas9 in three GSC cell lines, 387, CW468, and D456, using two sgRNAs (sg#2 and sg#3) (Fig. 2b) in order to validate the potential target. All three GSC lines demonstrated increased sensitivity to NK cell-mediated killing after the knockout (KO) of *CHMP2A* compared with the mock vector-only controls (mock vector is considered wild type (WT) for these studies) (Fig. 2a). To ensure this phenotype was not due to off-target effects of the sgRNAs, we silenced *CHMP2A* in the three cell lines using *CHMP2A*-specific shRNAs and observed similar increased sensitivity to NK cells in a killing assay (Supplemental Fig. 1a). As HNSCCs are known the be subject to NK cell infiltration, an increased number of NK cells within the tumor correlates with better survival^12^, to further confirm the role of CHMP2A in regulating NK cell-mediated killing we did a similar CRISPR-Cas9 mediated knockout of *CHMP2A* in three different HNSCC cell lines (Fig. 2d and Supplemental Fig. 1b): Cal27 (tongue squamous cell carcinoma), Detroit 568 (metastatic pharyngeal carcinoma), and HNSCC17B (laryngeal squamous cell carcinoma)^44^. We again observed a significant increase in NK cell-mediated cytotoxicity (Fig. 2c) in *CHMP2A*-KO cells. The KO of CHMP2A did not significantly impair the proliferation and viability of Cal27 cells (Supplemental Fig. 1c). Next, to confirm the increased sensitivity to NK cells we observed was indeed due to *CHMP2A*-KO, we overexpressed CHMP2A in Cal27 where CHMP2A had been previously knocked out (Fig. 2d). Rescuing CHMP2A expression restored resistance to NK cell-mediated killing, demonstrating that CHMP2A has a role in the resistance of Cal27 cells to NK cell-mediated killing (Fig. 2e). Although tumor cells can modulate their sensitivity to NK cells, we did not see any significant change in the expression of NK cell-activating or inhibitory ligands in Cal27 *CHMP2A*-WT (Supplemental Fig. 2a) and *CHMP2A*-KO cells (Supplemental Fig. 2b). Subsequently, we investigated The Cancer Genome Atlas (TCGA) database for GBM and HNSCC to evaluate a possible correlation between low *CHMP2A* expression and increased overall survival (Supplemental Fig. 3). We observed that low *CHMP2A* is associated with slightly increased (but not statistically significant) survival in GBM (Supplemental Fig. 3a), while the survival benefit of low *CHMP2A* expression is statistically significant in HNSCC (Supplemental Fig. 3b).

### CHMP2A increases NK cell migration via secretion of CXCL10 and CXCL12

**Fig. 3:**
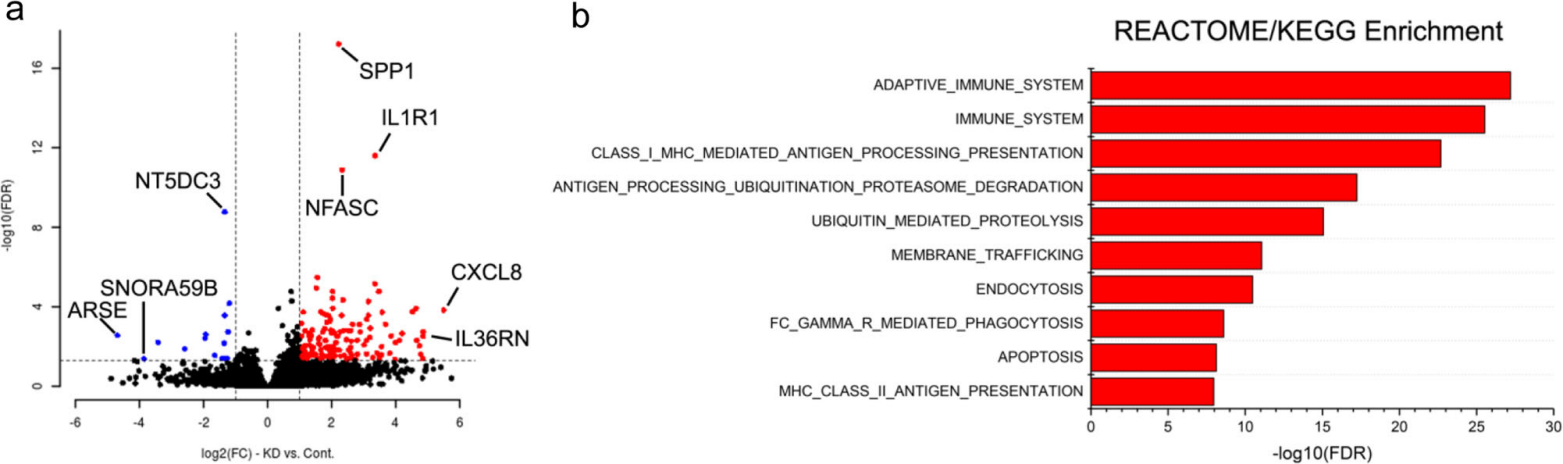
Gene expression analysis of *CHMP2A*-KO in GSCs. **a** Differential gene expression comparing *CHMP2A*-KO vs shCONT (*n* = 2 samples per cell line). Genes in red are upregulated with a knockout at log2 FC>1 and FDR<0.05, whereas genes in blue are downregulated with a knockout at log2 FC < −1 and FDR < 0.05. Top altered genes are labeled in the panel. **b** Reactome and KEGG gene set enrichment analysis of genes upregulated with CHMP2A KO. *Y* axis indicates −log10 FDR of the gene set enrichment.

**Fig. 4:**
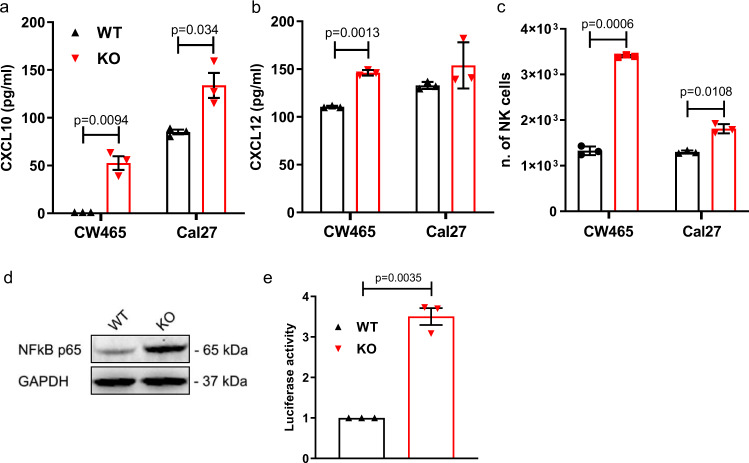
Secretion of CXCL10 and CXCL12 from *CHMP2A*-KO tumor cells increases NK cell migration. **a** ELISA comparing CXCL10 concentration WT and KO in CW468 and Cal27 cells. The error bars represent ±SEM across *n* = 3 independent experiments two-tailed Student’s *t* test was performed to determine statistical significance. **b** ELISA comparing CXCL10 concentration in WT and KO in CW468 and Cal27 cells. Error bars represent ±SEM from the mean across *n* = 3 independent experiments two-tailed Student’s *t* test was performed to determine statistical significance. **c** charts representing the number of NK cells migrating towards CW468-WT and KO (top chart) and Cal27-WT and KO (lower chart) cells analyzed by flow cytometry. Error bars represent ±SEM across *n* = 3 independent experiments. Two-tailed Student’s *t* test was performed to determine statistical significance. **d** Immunoblot analysis of NF-κB P65 in Cal27 CHMP2A-WT or KO. GAPDH has been used as a loading control. **e** Dual-luciferase reporter assay showing NF-κB activity in Cal27 CHMP2A-WT and KO cells. Error bars represent ±SEM from the mean across *n* = 3 replicates, two-tailed Student’s *t* test was performed to determine statistical significance. **d**, **e** Representative of *n* = 3 independent experiments.

To investigate potential mechanisms by which CHMP2A regulates NK cell-mediated killing, we analyzed the transcriptome of GSC cells by RNA sequencing. In 387 and CW468 cells, we found substantial differences in the expression of genes related to the tumor-immune system interaction, as well as membrane trafficking and endocytosis (Fig. 3). The most upregulated genes included cytokines, chemokines, and genes expressed during the innate immune response. Since tumor cells can modify their microenvironment and reduce the migration and antitumor activity of effector cells, including NK cells^45^, we were interested to identify CXCL10 and CXCL12 as increased in expression in the KO cells. We measured CXCL10 and CXCL12 produced by CW468 and CAL27-WT and *CHMP2A*-KO and found higher levels of CXCL10 and CXCL12 were secreted in the media when *CHMP2A* was deleted (Fig. 4a, b). Next, we assessed the migration of NK cells using *CHMP2A*-KO or WT cells as chemoattractants. An increase in NK cell migration was observed when NK cells were co-cultured with CW468 *CHMP2A*-KO and Cal27 *CHMP2A*-KO compared with WT cells (*p* < 0.01) (Fig. 4c). Interestingly we observed the upregulation of NF-κB P65 in Cal27 *CHMP2A*-KO by immunoblotting (Fig. 4d). It has been previously reported that NF-κB P65 activation can increase CXCL10 secretion^46,47^, therefore we used a reporter plasmid to further analyze NF-κB activity. We measured a three-fold increase in NFkB activity in Cal27 *CHMP2A*-KO compared with Cal27 *CHMP2A*-WT (Fig. 4e). Since ESCRT-III has been described to be a negative regulator of constitutive NF-κB activity by preventing the accumulation and the activation of NF-κB-inducing receptors^48^, further studies could elucidate the possible role of CHMP2A in regulating NF-κB signaling and the secretion of CXCL10.

### *CHMP2A* regulates tumor cell production of EVs

**Fig. 5:**
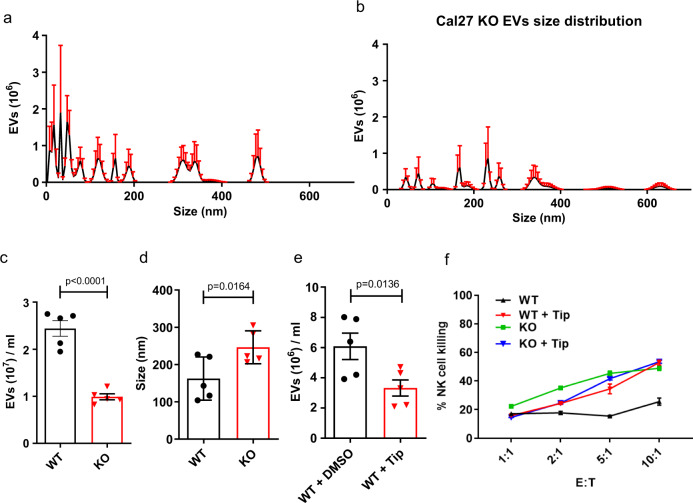
Cal27-derived EVs secretion is reduced in *CHMP2A*-KO cells and in tipifarnib treated WT Cal27 cells. **a**, **b** Charts showing the size distribution and number of EVs secreted from Cal27-WT (**a**) and KO (**b**) cells. Samples were loaded on a Nanosight LM10 and analyzed for 1 min for each of *n* = 5 technical replicates and the error bars represent ±SEM across *n* = 5. **c** comparison of EV number in Cal27-WT and KO calculated on 10 μl of EV suspension diluted in 1 ml of DPBS. **d** average EVs size analyzed during nanoparticle tracking of Cal27-WT and KO derived EVs. **e** comparison of EVs number in Cal27-WT treated with tipifarnib (Tip) and the corresponding DMSO control (CTRL) calculated on 10 μl of EVs suspension diluted in 1 ml of DPBS. **c**–**e** error bars represent ±SEM across *n* = 5 technical replicates and unpaired two-tailed Student’s *t* test was performed to determine statistically. **f** 4 hour cytotoxicity assay using NK cells as effectors against Cal27-WT, KO treated with tipifarnib or DMSO (CTRL). Error bars represent ±SEM, *n* = 3 replicates. Statistical analysis was performed by two-way ANOVA and Bonferroni’s post hoc multiple comparison test (comparing Cal27-WT vs Cal27-WT + Tip E:T, 10:1 *p* < 0.0001; 5:1 *p* < 0.0001; 2:1 and 1:1 ns). Data are shown in **a**–**f**, representative of *n* = 3 independent experiments.

We next evaluated the activity of NK cells when co-cultured in conditioned media from CW468 KO or WT cells culture. CW468-WT conditioned media reduced NK cell-mediated killing of CW468-WT compared with CW468 *CHMP2A*-KO-conditioned media cells (Supplemental Fig. 4a, b). NK cells also demonstrated less killing of Cal27-WT and *CHMP2A*-KO when cultured with WT conditioned media (Supplemental Fig. 4c, d). Since EVs can impair NK cell activity^28,29,32–34^, we tested if *CHMP2A-*KO could influence EV production from tumor cells by nanoparticle tracking^49^ (Fig. 5a, b and Supplemental Fig. 5a, b). We demonstrated that Cal27 *CHMP2A*-KO conditioned media had 59.5% fewer EVs than Cal27-WT (*p* < 0.0001) (Fig. 5c). In addition, we found EVs from KO cells were significantly bigger (*p* < 0.001) with an average size of 246.40 nm (SEM ± 19.70 nm) compared with 162.40 nm (± 25.90 nm) for Cal27-WT cells (Fig. 5d). The analysis on the GSC line CW468 showed comparable results. CW468 *CHMP2A-KO* conditioned media had fewer EVs than CW468-WT (Supplemental Fig. 5c), and the size of EVs from *CHMP2A*-KO cells was significantly larger (*p* < 0.05) than the EVs from WT CW468 (Supplemental Fig. 5d). To support the nanoparticle tracking results with visual imaging, we analyzed Cal27-WT and *CHMP2A-KO* EVs by TEM. Electronic microscopy showed sparse EVs, with no aggregates and overall bigger EVs in *CHMP2A-KO* samples (Supplemental Fig. 5f). CD9 is a glycoprotein of the tetraspanin family and is widely used as a marker for the characterization of EVs. Immunoblotting analysis of Cal27-WT and CHMP2A-KO EVs purified from the same volume of conditioned media showed reduced CD9 on KO samples, again demonstrating that CHMP2A-KO cells secrete fewer EVs than WT cells (Supplemental Fig. 5g). Since EVs secretion can be reduced using Tipifarnib, a farnesyl transferase inhibitor^50^, we treated Cal27-WT cells for 72 h with Tipifarnib and found a significantly lower number of EVs in treated cells compared to controls (*p* < 0.01) (Fig. 5e). Tipifarnib treated Cal27-WT cells also had increased sensitivity to NK cell-mediated killing comparable to Cal27 *CHMP2A-*KO cells (Fig. 5f), confirming our findings of CHMP2A’s role in mediating resistance to NK cells.

### Cal27-WT-derived EVs induce apoptosis in NK cells, limiting their antitumor activity

**Fig. 6:**
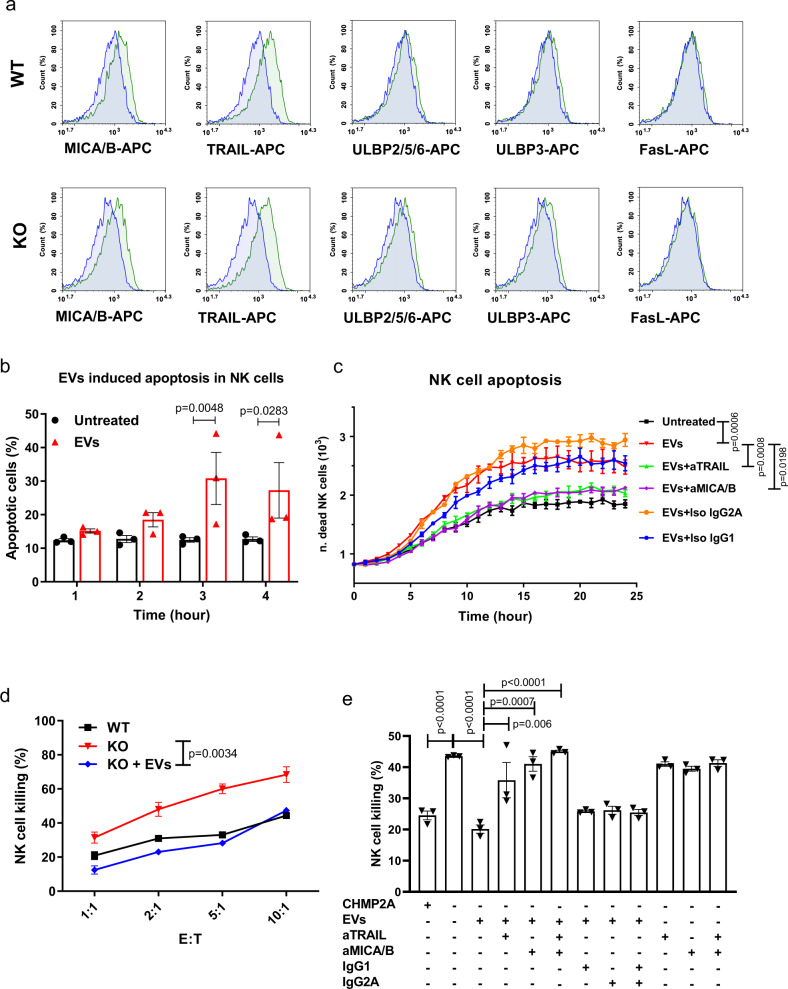
EVs secreted by Cal27 induce apoptosis in NK cells. **a** Cal27-WT and KO derived EVs were analyzed by flow cytometry for known NKG2D ligands MICA/B, ULBP2/5/6, ULPB3, and for TRAIL and FasL, proteins involved in intrinsic or extrinsic apoptosis pathway (green histograms). In blue is shown the isotype control. Representative of *n* = 2 independent experiments. **b** Flow cytometry CASP3/7 viability assay showing increasing NK cell death once exposed to EVs from Cal27 in a time course of 4-hours. The error bars represent ±SEM across *n* = 3 replicates. Two-way ANOVA and Bonferroni’s post hoc test was performed to determine statistical significance. Representative of *n* = 3 independent experiments. **c** 24 hour time course viability assay quantified using the IncuCyte real-time imaging system. *N* = 5 images/well taken for each of three replicates. The error bars represent ±SEM across *n* = 3 replicates. Statistical analysis was performed by two-way ANOVA and Bonferroni’s post hoc multiple comparison test. Representative of *n* = 2 independent experiments. **d** 4 hour cytotoxicity assay using NK cells as effectors against Cal27-WT or KO as target cells. Here we show how Cal27-derived EVs reduce the killing in Cal27-KO. Error bars represent ±SEM across *n* = 3 replicates. Statistical analysis was performed by one-way ANOVA and Bonferroni’s post hoc test (ns, not significant). **e** antibodies blocking MICA/B, TRAIL, or a combination of the two (aMICA/B and aTRAIL) limit the inhibitory effect of EV on NK cell-mediated killing. 4-hour cytotoxicity assay using NK cells as effectors against Cal27-WT or KO as target cells (5:1) incubated with Cal27-WT-derived EV and with MICA/B and TRAIL blocking antibodies or respective isotypes controls. Error bars represent ±SEM across *n* = 3 replicates. Statistical analysis was performed by two-way ANOVA and Bonferroni’s post hoc multiple comparison test. **d**–**e** Representative of *n* = 2 independent experiments.

To further investigate the effect of EVs on NK cells, we analyzed EVs from WT and *CHMP2A*-KO Cal27 cells by flow cytometry for the expression of MICA/B, TRAIL, FasL, and ULBPs. Although no differences in expression of these markers were observed between Cal27-WT and KO-derived EVs (Fig. 6a), we found EVs expressed NKG2D ligands MICA/B, and TRAIL. Notably, previous studies demonstrate EVs containing MICA/B can inhibit NK cell activity^28,29^ and EVs-bearing TRAIL can induce apoptosis in NK cells^32–34^. Consequently, since NK cells express NKG2D and TRAIL receptors (TRAIL-R1 and TRAIL-R2) (Supplemental Fig. 6a), we hypothesized Cal27-derived EVs could reduce NK cell viability. NK cells were incubated with Cal27-derived EVs and assessed viability through the activation of the Caspase-3–7 (Casp3–7) pathway. EVs derived from Cal27 cells significantly increased NK cell death after 3 hour of treatment compared with untreated NK cells (Fig. 6b). Subsequently, we incubated NK cells with EVs for 24-h, supplemented with MICA/B or TRAIL blocking antibodies that led to significantly less cell death than NK cells incubated with EVs only or EVs incubated with the matching isotype control antibodies (Fig. 6c) (in Supplemental Fig. 6b we report the data from the first 5 hours as compared with the data displayed in Fig. 6b). These results demonstrate that Cal27-derived EVs-bearing MICA/B and TRAIL can induce apoptosis in NK cells. Next, to determine whether the EVs were increasing the resistance of Cal27-KO cells to NK cells we co-cultured NK cells with Cal27 *CHMP2A*-KO cells and EVs. Cal27 *CHMP2A*-KO showed significantly reduced sensitivity to NK cell killing when incubated together with EVs (*p* < 0.01), comparable with WT levels (Fig. 6d). Similarly, to Cal27, EVs significantly increased the viability of CW468 reducing the killing activity of NK cells (Supplemental Fig. 5e). Treatment with MICA/B and TRAIL blocking antibodies significantly reduced the inhibiting effect of EVs when NK cells were co-cultured with KO Cal27 cells plus EVs (Fig. 6e). A combination of the two antibodies further improved the killing of the NK cells (Fig. 6e). In addition, we demonstrated these EVs reduced NK cell-mediated killing of K562 cells, an NK cell-sensitive cell line, and this inhibition was rescued by blocking MICA/B or TRAIL (Supplemental Fig. 6c). As shown in Fig. 6a, EVs secreted by tumor cells are composed of small (SEV) and large (LEV) EV. We separated through a gradient of ultracentrifugation LEV from SEV and co-cultured with NK cells for four hours. Interestingly SEV significantly increased the number of apoptotic NK cells while LEV did not. A combination of SEV and LEV enhanced apoptosis in NK cells (Supplemental Fig. 6d). To further confirm the role of SEV in impairing NK cell-mediated killing we performed a killing assay coculturing Cal27 *CHMP2A*-KO cells with SEV, LEV, or a combination of the two (Supplemental Fig. 6e). SEV reduced the killing of NK cells confirming their role in inhibiting NK cell killing of Cal27. Together these results demonstrate that Cal27 cells utilize EVs as a mechanism to increase resistance to NK cell-mediated cytotoxicity through the induction of NK cell death.

### *CHMP2A*-KO increases tumor cell sensitivity to NK cell-mediated killing in an in vivo HNSCC xenograft mouse model

**Fig. 7:**
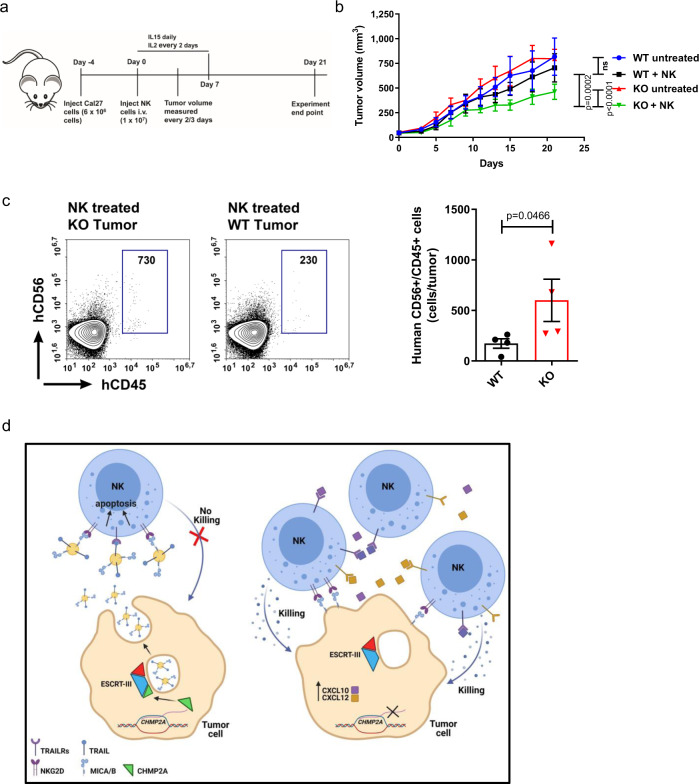
CHMP2A-KO HNSCC xenograft model shows increased sensitivity to NK cells. **a** Schematic of in vivo treatment. Non-obese diabetic/severe combined immunodeficiency/γc^−/−^ (NSG) mice were inoculated subcutaneously with 6 × 10^6^ cells (Cal27-WT or KO) and injected i.v. with 1 × 10^7^ NK cells. NK cells were supported by injections IL-15 daily and IL-2 every other day for 1 week. Tumor volume was monitored every 2–3 days. **b** Tumor volume progression over 21 days shown as mean ±SEM of *n* = 5 mice per treatment. Statistical analysis was performed by two-way ANOVA and Bonferroni’s post hoc multiple comparison test (ns, not significant). **c** Representative flow cytometry dot plots showing increased NK cell infiltration in Cal27 *CHMP2A* KO tumors. The gating strategy has been defined by combining NK cells and a cell suspension from the untreated tumor. Tumor samples without NK cell treatment were mixed with 2 × 10^4^ human NK cells, stained with anti-human CD45 and anti-human CD56 antibodies or isotype controls, and analyzed. Cells double positive for (human) hCD45 and hCD56 were gated and the gate was used to quantify the number of infiltrating NK cells in Cal27-WT and CHMP2A KO tumors. The graph shows the average NK cell infiltration for all mice in each group (*n* = 4 mice per group). Error bars represent ±SEM across *n* = 4 technical replicates and an unpaired one-tailed Student’s *t* test was performed to determine statistical significance. **d** Cartoon describing the proposed mechanism of CHMP2A KO in tumor cells. Tumor cells secrete EVs-bearing ligands like MICA/B and TRAIL, which suppress the antitumor effect of NK cells by binding to NKG2D and TRAIL-R to induce apoptosis on NK cells. By CHMP2A KO we reduce EVs secretion from tumor cells decreasing their inhibitory function. Tumor cells CHMP2A KO also secrete CXCL10 and CXCL12, chemokines that increase NK cell migration culminating in enhanced NK cell-mediated cytotoxicity (the cartoon has been created with Biorender.com).

To evaluate whether KO has a similar impact on tumor sensitivity to NK cells in vivo, we established an HNSCC xenograft mouse model. CHMP2A-WT or KO Cal27 cells injected into immunodeficient mice showed no significant difference in growth. After tumors reached 50 mm^3^ in volume, human NK cells were injected intravenously (i.v.) (schematic in Fig. 7a). Notably, Cal27-KO cells were markedly more sensitive to NK cell-mediated killing in vivo than Cal27-WT cells (Fig. 7b). NK cell-treated mice bearing Cal27-KO tumors showed a significant reduction in tumor volume compared with untreated Cal27-KO control while mice with Cal27-WT tumors had no significant decrease in tumor volume compared to untreated Cal27-WT mice (Fig. 7b). Flow cytometric analysis of tumor-infiltrating NK cells demonstrates a significant increase of human NK cells in *CHMP2A-KO* tumors (Fig. 7c), suggesting that reducing the EVs secretion in the tumor environment can increase the number of tumor-infiltrating NK cells. These data confirmed the role of CHMP2A to promote resistance of HNSCC Cal27 cells to NK cell-mediated killing in vivo.

### NK cell-specific signature correlates with low *CHMP2A* expression in GBM and HNSCC

NK cells, as part of innate immunity, are the first line of defense against tumors. Tumors often develop an immune-suppressive TME leading to inhibition of cytotoxic T lymphocytes and NK cells. GBM and HNSCC show completely different NK cells infiltration numbers. NK cells are among the least abundant population of all immune cells that infiltrate GBM^51^, whereas NK cells are known to infiltrate HNSCC and a high degree of NK cells infiltration positively correlates with HNSCC survival^12^. Even though GBM cells can express inhibitory ligands for NK cells (i.e., HLA class I), they can express activating ligands for NKG2D^51^, therefore, increasing the number of active NK cells in GBM could be beneficial. Novershtern and colleagues described a signature for immune cells during their differentiation process to maturation, including a group of genes upregulated specifically in mature NK cells^52^. We matched the top 20 genes in Novershtern data set for NK cells (NK 20) with low or high expression of *CHMP2A* in GBM and HNSCC (Supplemental Fig. 7). We found a positive correlation between low *CHMP2A* expression and the NK cell signature. These results support a model whereby CHMP2A increases resistance to NK cells in GBM and HNSCC, reducing the antitumor activity of NK cells.

## Discussion

NK cells are a key part of the innate immune system and play an important role in controlling the development of malignancies^1,3^. However, solid tumors are generally less sensitive than hematological malignancies to NK cell-mediated killing, at least in part owing to the immune inhibitory properties of the TME^53–55^. Frequently, the tumor cells halt immune cells migration or inhibit their cytolytic functions, reducing the efficacy of these treatments^56^.

CRISPR-Cas9 screens have been used recently to discover new protein targets or druggable molecular pathways in tumor cells^57–59^ and combined with effector cells in TCT screenings^7–9^. In their study, Pech et al.^9^ used an NK cell-based TCT to identify DDB1-and-Cul4-associated factor 15 (DCAF15) as a novel regulator of NK cell-mediated killing. Notably, this work used the NK92 cell line as effector cells and the highly NK cell-sensitive erythroleukemia K562 cell line as the target cell population. It is possible that these findings using an aneuploid NK cell line and the very NK cell-sensitive K562 cells do not fully reflect the diversity of mechanisms that NK cells use to engage and kill tumor cells, especially solid tumors that are typically more resistant to NK cell activity. More recently a whole-genome CRISPR screen has been combined with profiling relative inhibition simultaneously in mixtures (PRISM) approach to study the response versus the resistance of several solid tumor cell lines to NK cells^60^. B7-H6 upregulation was found to be an important gene associated with marked NK cell sensitivity while HLA-E KO was associated with increased sensitivity to NK cell cytotoxicity. Although not present in the top 20 hits, we also observed HLA-E as an important negative regulator of response to NK cells in our CRISPR screen (Fig. 1d), altogether with other MHC class I genes and genes involved in surface expression of MHC I molecules.

Using a CRISPR-Cas9 TCT screen we identified CHMP2A, a component of the ESCRT-III complex, as a regulator of GSC and HNSCC cell sensitivity to NK cell-mediated cytotoxicity. We observed increased migration of NK cells towards CHMP2A KO tumor cells that showed increased secretion of CXCL10 and CXCL12, chemokines involved in NK cell migration^39,45^. Our findings are also supported by a bioinformatic analysis of the TCGA database for GBM and HNSCC describing an inverse correlation between *CHMP2A* expression and the NK cell signature in tumors. That is, there is higher NK cell infiltration of tumors that have lower CHMP2A expression.

Notably, the polyprotein ESCRT-III that contains CHMP2A as a subunit is involved in the last phase of cell membrane deformation and fission^61,62^, a process required for EVs budding^42^. Although some studies have suggested that the ESCRT-III complex can mediate tumor cell resistance to killing, more precise mechanisms have been unclear^63^. EVs have been characterized to impair the activation of immune cells within tumors^25–29,34,35^. In patients with HNSCC, EVs are associated with the suppression of lymphocytes, (including NK cells), and with disease stage and activity^16^. In GBM, EVs are involved in inducing immune-tolerance through the activation of myeloid-derived suppressor cells^64^. More in general EVs are considered to play a relevant role in creating an immune-suppressive microenvironment in GBM and HNSCC^14,65^. Moreover, it has been recently described that gain of function TP53 can be transferred from tumor cells to normal fibroblast via EV, resulting in their conversion to cancer-associated fibroblasts^66^. Here, we observed a correlation between reducing EVs secretion in GSC and HNSCC cells and the increased sensitivity to NK cell killing both in vitro and in vivo. Focusing on the mechanisms by which EVs promote tumor resistance, EVs carrying NKG2D ligands were shown to induce a downregulation of cell surface NKG2D in both NK and T cells^25,27^ or act as decoys^26^. Blocking the shedding of NKG2D ligands can increase NK cell-antitumor activity^31^. Nevertheless, not only MICA/B are involved in this resistance mechanism, and recently Sharma and colleagues showed EVs expressing TRAIL can impair NK cell functions^34^. Our data support the idea that EVs may express various ligands for NK cell receptors that can affect their effector functions, thus blocking the binding of MICA/B or TRAIL on EVs can improve NK cell-mediated cytotoxicity. These findings suggest CHMP2A and more widely ESCRT-III being part of the key immune-suppressive mechanism that involves secretion of tumor EVs that can inhibit NK cell-mediated killing, including inducing apoptosis in NK cells. Therefore, tumor-derived EVs may not only be used as a reliable biomarker to characterize tumors^23^ but also considered to better define the immune escape mechanisms used by different tumors and studied to develop new strategies to overcome tumor resistance to immune cells.

In conclusion, we have used a TCT CRISPR screen to discover a key gene that regulates tumor sensitivity to NK cells. This study provides an explanation of a mechanism that contributes to resistance in GBM and HNSCC to NK cell-mediated killing. Our data demonstrate CHMP2A and tumor secreted EVs can be responsible for inducing apoptosis in NK cells, thus limiting their cytotoxic potential. The evaluation of the expression of CHMP2A and characterization of tumor secreted EVs in patients may explain some mechanisms of immune escape and speed the development of new drugs that can target these immune-suppressive processes.

## Methods

### All studies comply with local and national ethical guidelines

Animal studies were approved by the University of California, San Diego Institutional Animal Care and Use Committee and followed the National Institutes of Health’s Guide for the Care and Use of Laboratory Animals. All studies with biological agents were approved by the University of California, San Diego Institutional Biosafety Committee.

### Lentiviral transduction on GSCs

The sgRNA library and single-targeted sgRNA lentiviral plasmids for GSC transduction were purchased from Addgene (#73179 and #52961, respectively). Lentiviral particles were generated as previously described^67^. For lentiviral transduction, GSC tumorspheres were dissociated into single cells using Accutase (Innovative Cell Technologies), resuspended in GSC media, and optimal lentivirus was added in order to achieve 30–40% infection efficiency. GSCs were then washed once after 12 h, resuspended in fresh GSC media, and cultured for 2 days. Transduced cells were enriched by puromycin (Thermo Fisher Scientific, final concentration 1 μg/ml) selection for seven continuous days.

### CRISPR screening on GSCs

GSCs transduced with the CRISPR KO library were dissociated into single cells and co-cultured with NK cells at an E:T ratio of 2:1 in culture plates pre-coated with Matrigel (Corning). After 24 h, the media containing NK cells and tumor debris was removed and the remaining GSCs were washed with phosphate-buffered saline (PBS) and harvested. Genomic DNA was isolated from the remaining GSCs after co-culture with NK cells, as well as GSCs harvested before co-culture and GSCs after monoculture for 24 h.

### CRISPR-Cas9 screen analysis

FASTQ files were trimmed to 20 bp CRISPR guide sequences using BBDuk from the BBMap (B. Bushnell) toolkit and quality control as performed using FastQC (Babraham Bioinformatics). FASTQs were aligned to the library and processed into counts using the MAGECK “count” function^43^. Genes were then filtered such that the sum of normalized counts >10. Hits were ranked by RIGER using the Kolmogorov–Smirnov algorithm.

### RNA-seq analysis

RNA sequencing analysis was performed as previously described^10^. Briefly, the total mRNA from GSC shCHMP2A-KO and shCONT was isolated and purified by the RNeasy Mini Kit (Qiagen Inc.) and sequenced with Illumina protocols on a HiSeq 2500 to generate 50-bp reads. Trim Galore (https://www.bioinformatics.babraham.ac.uk/projects/trim_galore; RRID:SCR_011847) was used to trim adaptors and remove low-quality reads. Reads were quantified against Gencode v29 using Salmon (https://combine-lab.github.io/salmon/; RRID:SCR_017036) with correction for fragment-level GC bias, positional bias, and sequence-specific bias. Transcripts were summarized to gene level and processed to transcripts per million (TPM) using the R/Bioconductor (https://www.bioconductor.org/) package DESeq2 (https://bioconductor.org/packages/release/bio-c/html/DESeq2.html; RRID:SCR_000154). Comparisons were performed using contrasts in DESeq2 followed by Benjamini–Hochberg adjustment to correct for false discovery rate (FDR).

### Reactome networks and KEGG pathways

As described in Wang et al.^10^ Reactome networks were derived from RNA-seq data using the Cytoscape Reactome FI plugin (RRID:SCR_003032). Genes upregulated with a knockout at log2 FC > 1 and FDR < 0.05 and genes downregulated with a knockout at log2 FC < −1 and FDR < 0.05, plus the target gene were input into Reactome FI, and all genes with at least one edge were included in the network plot. Pathway enrichment was performed on this network of genes using the Reactome FI enrichment option. Box plots for genes from selected pathways were generated using RNA-seq TPM data. KEGG pathway visualizations were generated using the R/Bioconductor package pathview (https://www.bioconductor.org/packages/release/bioc/html/pathview.html) for selected pathways.

### Cell culture

GSC lines 387, CW468, D456, and 1517^68–70^ were cultured in phenol red-free Neurobasal media plus B27 supplement (Invitrogen), supplemented with 2 mM l-glutamine (Invitrogen), 1 mM sodium pyruvate (Invitrogen), 10 ng/ml basic fibroblast growth factor, 10 ng/ml epidermal growth factor (EGF) (R&D Systems). Cal27, Detroit568 and UMSCC17B were cultured in Dulbecco’s Modified Eagle Medium (DMEM)-F12 1:1 (Gibco), 10% fetal bovine serum (FBS), supplemented with 2 mM l-glutamine (Invitrogen), 0.1 mM MEM non-essential amino acids (NEAA) (Invitrogen) (HNSCC complete media). Cal27-WT and KO cells were cultured in serum-free media (SFM) Defined Keratinocyte SFM (Invitrogen) supplemented with 2 mM l-glutamine (Invitrogen), 0.1 mM NEAA (Invitrogen) and 2 ng/ml EGF (R&D Systems) (Cal27 SFM). HEK 293FT cells were cultured in DMEM (Invitrogen), 10% FBS supplemented by 0.1 mM NEAA, 2 mM l-glutamine, 1 mM MEM Sodium Pyruvate, 1% PenStrep and 500 μg/ml Geneticin (Invitrogen).

### Generation of CHMP2A KO cell lines using lentiviral vectors

Four sgRNA targeting *CHMP2A* were obtained from the whole-genome CRISPR library (Addgene #73179) and cloned into the sgRNA-Cas9 lentiviral vector (Addgene #52961). sgRNA#1—AGACGCCAGAGGAGCTACTG, sgRNA#2—CAAACTTGCGCACATAGCGC, sgRNA#3—TCGATGGCACAAGCCATGAA, sgRNA#4—TCTCTAGTTTCTGTCGCTCG. HEK 293FT (4 × 10^6^) were seeded the day before transfection in a 100 mm^2^ culture dish. 24 h later cells were co-transfected with sgRNA-Cas9 lentiviral vector (5 μg) or shRNA plasmids (Sigma), pPAX (3.5 μg) (Addgene), and pMD2.G (1.5 μg) (Addgene) using LipoD293 (SignaGen Laboratories) following manufacturer’s protocol. Lentiviral supernatant was collected 48 h post transfection, filtered 0.45 μm, and concentrated using Lenti-X concentrator solution (Takara Bio). Recipient cells once reached 40–50% confluency, were infected and incubated at 37 °C ON with viral supernatant containing 10 μg/ml polybrene (EMD Millipore) and replaced 24 h later with fresh media. After 48 h, transduced cells were cultured in fresh media containing 1 μg/ml puromycin (EMD Millipore) for 2–3 days. Single clone selection was performed in Cal27 mock and KO cells seeding cells at limiting dilution after drug selection. Single clones were identified by sequencing PCR products containing amplified DNA nearby the sgRNA binding site. sgRNA#3—TCGATGGCACAAGCCATGAA has been used to generate Cal27-KO cells.

### Isolation and expansion of NK cells

Peripheral blood mononuclear cells (PBMC) were isolated through density gradient centrifugation from an apheresis product (obtained from the San Diego Blood Bank Center) and NK cells were sorted using EasySep Human NK Cell Enrichment Kit (StemCell Technologies) which depletes CD3^+^ and CD19^+^ cells. The use of PBMC from anonymized donors was approved by the Committee on the Use of Human Subjects in Research at the University of California, San Diego. Peripheral blood NK cells were cultured in RPMI 1640 (Invitrogen), 10% heat-inactivated FBS, 2 mM L-glutamine, 1% PenStrep and supplemented with 50 IU/mL of hIL-2 every three days or media change. NK cells were co-cultured once a week with irradiated K562-mbIL-21-4-1BBL artificial antigen-presenting cells kindly provided by Dr. Dean A. Lee (Nationwide Children’s Hospital) for expansion^71^.

### Generation of CHMP2A overexpressing Cal27

In all, 8 × 10^5^ cells Cal27-KO were transfected with CHMP2A (NM_014453) Human Tagged ORF Clone (Origene) via nucleofection using Amaxa 2D (Lonza) and Cell Line Nucleofector Kit V (Lonza) following manufacturer’s protocol. After nucleofection cells were seeded in six-well plates and 24 h later media was changed and supplemented with Geneticin (1 μg/ml) (Invitrogen) for selection. CHMP2A overexpression was assessed by immunoblotting.

### Immunoblotting

Immunoblotting was performed according to Invitrogen protocols for Mini Gel Tank and iBlot2 dry system. In brief, cell lysates were prepared to incubate cells with RIPA buffer (Invitrogen) containing Halt Protease Inhibitor Cocktail (Invitrogen) and quantified using BCA assay (Thermo Fisher Scientific). EVs lysates were prepared incubating cells with 50 μl of RIPA buffer (Invitrogen) containing Halt Protease Inhibitor Cocktail (Invitrogen) and sonicated for 10 min. In all, 25 μl of EVs lysate was loaded on the gel. Proteins were separated using NuPAGE Bis-Tris 4–12% gels (Invitrogen) and transferred to nitrocellulose membranes using the iBlot2 dry method. Membraned were blocked for 40 min in 4% milk and incubated with the primary antibodies in 4% milk 1.5 h at room temperature for anti-CHMP2A antibody or ON at 4 °C for all the other antibodies used. After incubating the membrane with the appropriate secondary antibody conjugated to horseradish peroxidase, protein levels were detected using Immobilon Western Chemiluminescent HRP Substrate (EMD Millipore). CHMP2A overexpression lysate (OL) control used in Fig. 2c was purchased from Origene. CHMP2A Antibody, rabbit polyclonal, Proteintech, cat. no. 10477-1-AP (1:600). GAPDH Loading Control Monoclonal Antibody (GA1R) (1:1000), Invitrogen, cat. no. MA5-15738. CD9, clone (D8O1A) Rabbit mAb, Cell Signaling, cat. no. 13174S (1:1000).

### Tipifarnib treatment

Cal27-WT and CHMP2A KO cells were treated with tipifarnib as follows: prior to performing NK cells killing assays, cells were trypsinized and harvested by centrifugation at 400 × *g*, pellet resuspended, cells seeded at 1 × 10^5^ cells/ml in HNSCC compete for media and treated with tipifarnib 1 μM^50^ or DMSO as a control for 72 h. Prior to collection and analysis of cell-derived EVs, Cal27 were trypsinized and harvested by centrifugation at 400 × *g*, cells washed in 5 ml of 0.22 μm filtered sterile Dulbecco’s phosphate-buffered saline (DPBS) and harvested by centrifugation. Pellet was resuspended in Cal27 SFM, 5 × 10^6^ cells plated in 15 ml of Cal27 SFM in a T75 flask and treated with tipifarnib 1 μM or DMSO as control for 72 h.

### Antibodies used

The following antibodies were used for flow cytometry (all anti-human unless indicated):

PE-Cy7 anti-human CD56 mIgG1#, BioLegend, clone HCD56, cat. n. 318318 (1:100)

APC anti-human CD45, mIgG1#, BD Biosciences, clone H130, cat. n. 555485 (1:50)

APC anti-human MICA/MICB Antibody, Biologend, cat. n. 320907 (1:100)

Human TRAIL-R2/TNFRSF10B PE-conjugated Antibody - R&D, cat. n. FAB6311P (1:100)

PE anti-human ULBP2/5/6, R&D, Clone: 165903, cat. n. FAB1298P (1:100)

Human ULBP3 Alexa Fluor® 647-conjugated Antibody, R&D, cat. n. FAB1517R (1:100)

PE anti-hCD178 (FasL), eBioscience, cat. n. 12-9919-42 (1:100)

PE Mouse Anti-Human CD112, BD Biosciences, cat. n. 551057 (1:50)

PE Mouse Anti-Human HLA-ABC, BD Biosciences, cat. n. 557349 (1:50)

PE anti-CD155 antibody, Biolegend, cat. n. 337609 (1:100)

PE anti-HLA-E antibody, Biologend, cat. n. 342603 (1:100)

APC anti-human CD274 (B7-H1, PD-L1) Antibody [Clone: 29E.2A3], Biolegend, cat. n. 329707 (1:100)

PE anti-human CD276 (B7-H3) Antibody [Clone: MIH42], Biolegend, cat. n. 351003 (1:100)

PE anti-human CD262 (DR5, TRAIL-R2) Antibody [Clone: DJR2-4 (7-8)], Biolegend, cat. n. 307405 (1:100)

APC anti-human CD261 (DR4, TRAIL-R1) Antibody [Clone: DJR1], Biolegend, cat. n. 307207 (1:100)

APC Mouse Anti-Human CD314 (NKG2D), BD Biosciences, cat. n. 558071 (1:100)

### Fluorescence-activated cell sorting (FACS)

All the flow cytometry-based assays were performed on a NovoCyte (ACEA Biosciences) and data were analyzed using NovoExpress software. Cal27 cells were trypsinized and centrifuged at 400 × *g* for 5 min while NK cells resuspended in media and centrifuged at 400 × *g* for 5 min. Cells were resuspended in DPBS + 2% FBS (flow buffer) and stained with trypan blue, counted using an inverted microscope, and 1 × 10^5^ cells were dispensed per sample. Cells were incubated on ice in the dark for 20 min in a flow buffer containing the antibodies of interest. Stained cells were centrifuged at 400 × *g* for 5 min and washed flow buffer two times. Finally, cells were resuspended in 300 μl of flow buffer containing SYTOX Blue Dead Cell Stain (Life Technologies) diluted by a factor of 1000 and analyzed by FACS.

### Flow cytometry NK cells killing assay

Target cells were pre-stained with CellTrace Violet (Thermo Fisher Scientific) at a final concentration of 5 mM in PBS for 15 min at 37 °C. Cells were incubated in complete culture medium containing FBS for 5 min and harvested by centrifugation. Cells were resuspended in culture media prior to being mixed with NK cell cultures at the indicated effector to target (E:T) ratios. When performing EVs or anti-MICA/B and anti-TRAIL blocking treatments, reagents or EVs were added to the specific wells in culture media plus an Fc Receptor Binding Inhibitor (Thermo Fisher Scientific), 20 μl/test. Co-cultures were incubated at 37 °C and after 3.5 h CellEvent Caspase-3/7 Green Detection Reagent (Thermo Fisher Scientific) was added for an additional 30 min of culture for a total incubation time of 4 h. SYTOX AADvanced dead cell stain solution (Thermo Fisher Scientific) was added during the final 5 min of staining. Cells were then analyzed by flow cytometry. NK cells killing was calculated by subtracting the background of untreated target cells from all the other samples of the same experimental group. Experiments were performed with three independent biological triplicates. To perform the killing assay described in Supplemental Fig. 4, CW468-WT and KO cells were cultured for three days, and media was collected after 5 min centrifugation at 400 × *g* to remove dead cells. Target cells and NK cells were co-cultured on conditioned media and the killing assay was performed and analyzed as described above.

### Flow cytometry Caspase-3/7 apoptosis assay

NK cells were pre-stained with CellTrace Violet (Thermo Fisher Scientific) at a final concentration of 5 mM in PBS for 15 min at 37 °C. Cells were incubated in a complete culture medium containing FBS for 5 min and harvested by centrifugation. NK cells were resuspended in media containing CellEvent Caspase-3/7 Green Detection Reagent and SYTOX AADvanced dead cell stain solution and treated with Cal27-derived EVs and incubated for 4 h. Treated cells and the corresponding untreated controls were collected every hour by flow cytometry. Experiments were performed with three independent biological triplicates.

### IncuCyte Caspase-3/7 apoptosis assay

NK cells were pre-stained with CellTrace Far Red (Thermo Fisher Scientific) at a final concentration of 5 mM in PBS for 15 min at 37 °C. Cells were incubated in complete culture medium containing FBS for 5 min and harvested by centrifugation. NK cells were resuspended in media containing IncuCyte Caspase-3/7 Green Apoptosis Assay Reagent (Essen Bioscience) diluted by a factor of 1000 and 1 × 10^4^ NK were seeded in a 96 well plate previously coated with Poli-d Lysine (Sigma-Aldrich) ON at 37 °C. depending on treatments combinations Cal27-derived EVs, anti-MICA/B, anti-TRAIL, mouse IgG1, and IgG2A isotypes were mixed in culturing media and added to NK cells. Cells were centrifuged for 5 min at 400 × *g* to let NK cells adhere to the coated well monitored on the IncuCyte ZOOM to acquire images every 1 h for 24 h. Experiments were performed with 3 independent biological triplicates. The apoptosis was quantified by analyzing the overlay of Caspase-3/7 (green) within the red labeled NK cells.

### Transwell migration assay

WT and KO cells were seeded at 1 × 10^5^ cell/well in a 24 well plate in 600 μl SFM. Two days later 1 × 10^5^ NK cells were added in 200 μL SFM to the upper chamber (5 μm pore size), and the plates were incubated for 3 h at 37 °C. Cells in 300 μl of media in the lower chamber were incubated with NKG2D antibody or mouse IgG isotype to discriminate NK cells from any floating tumor cells and after 30 min NK cells number was determined by flow cytometry counting cells in 200 μl of media using a NovoCyte flow cytometer. Data are presented as a number of NK cells calculated in 600 μl of media. The typical NK cells marker CD56 (NCAM) was not used to detect NK cells because expressed on GSC (data not shown).

### Chemokine analysis

CW468 and Cal27 cell lines supernatants were generated by culturing 1 × 10^5^ cells/ml in 24 well plates in SFM for 48 h. Dead cells were removed by 5 min centrifugation at 400 × *g* and the supernatants were analyzed for CXCL10 and CXCL12 using IP-10 (CXCL10) Human ELISA Kit (Invitrogen) and ELISA, Human CXCL12/SDF-1 DuoSet, (R&D Systems) according to the manufacturer’s protocols on Infinite 200Pro (Tecan). Experiments were performed in duplicate and repeated three times.

### NF-κB reporter assay

pSI-Check2-hRluc-NFkB-firefly reporter plasmid^72^ was a gift from Qing Deng (Addgene plasmid # 106979). In brief, Cal27 CHMP2A-WT or KO cells were electroporated with 1 μg of the reporter plasmid via nucleofection using Amaxa 2D (Lonza) and Cell Line Nucleofector Kit V (Lonza) following manufacturer’s protocol and plated in 12-well plates. After 48 h, cells were lysed, and luciferase activity was measured using the Dual Glo-Luciferase assay system (Promega) on Infinite 200Pro (Tecan). The firefly luciferase activity was normalized to the renilla luciferase activity and the ratio of renilla luciferase activity to firefly luciferase activity of Cal27 CHMP2A-WT was set as 1. Experiments were performed in triplicates and repeated two times.

### EVs harvesting and tracking analysis

At day 1, WT or *CHMP2A*-KO cells were harvested accordingly to the cell type, pellet resuspended, and cells washed in 5 ml of 0.22 μm filtered sterile DPBS and harvested by centrifugation. Cal27 and CW468 were resuspended in ±SEM and 5 × 10^6^ cells plated in 15 ml of SFM (Neurobasal media for GSC or Cal27 SFM for Cal27) in T75 flasks and cultured at 37 °C for 3 days. At day 3, EVs were collected following the manufacturer’s protocol. In brief, conditioned media was centrifuged at 3000 × *g* for 15 min to remove dead cells and apoptotic bodies. 1:5 ExoQuick ULTRA EVs Isolation Kit for Tissue Culture Media (SBI System Biosciences) was added to collected media, gently mixed, and incubated at 4 °C ON. The following day the suspension was centrifuged at 3000 × *g* for 10 min, the supernatant removed, and EVs were resuspended in 0.22 μm filtered sterile DPBS and vortexed for 1 min. For EVs tracking analysis, 10 μl of EVs suspension were diluted in 1 ml of 0.22 μm filtered sterile DPBS and analyzed on a NanoSight LM10.

### SEV and LEV separation through ultracentrifugation

At day 1 Cal27-WT cells were harvested, pellet resuspended, and cells washed in 5 ml of 0.22 μm filtered sterile DPBS and harvested by centrifugation. Cal27 were resuspended in ±SEM and 5 × 10^6^ cells plated in 15 ml of SFM in T75 flasks and cultured at 37 °C for 3 days. At day 3, media was collected and centrifuged at 2000 × *g* for 10 min to remove dead cells. The pellet was discarded, and the supernatant ultracentrifuged at 10,000 × *g* for 30 min. After centrifugation, the supernatant was collected, and the pellet that contains LEV resuspended in 0.22 μm filtered sterile DPBS. The supernatant was further ultracentrifuged at 100,000 × *g* for 2 h to collect SEV. After ultracentrifugation, the supernatant was gently removed and the pellet of SEV resuspended in 0.22 μm filtered sterile DPBS.

### Flow cytometric analysis of EVs

On Day 1, freshly isolated Cal27-derived EVs collected as described in the paragraph above were stained using PKH26 Red Fluorescent Cell Linker Kit (Sigma-Aldrich) following a slightly modified manufacturer’s protocol. EVs were resuspended in Diluent C, mixed with PKH26, and incubated for 5 min at RT. PKH26 was quenched with 2 ml of 0,22 μm filtered sterile DPBS + 10% BSA bring the volume up to 8.5 mL in SFM. ExoQuick ULTRA EVs Isolation Kit solution was added at 1:5 ratio, gently mixed, and incubated at 4 °C ON. On day 2 EVs were harvested by centrifugation at 3000 × *g* for 10 min and resuspended in 0.22 μm filtered sterile DPBS + 1% BSA (EVs flow buffer) and distributed in 100 μl volume. EVs were incubated as single staining with MICA/B, TRAIL, ULBP3, ULBP2/5/6, FasL antibodies, and matching isotypes for 30 min on ice and diluted with 200 μl of EVs flow buffer. Samples were analyzed by flow cytometry gating the red fluorescent particles and measuring specific antigen expression. Due to the nanometer size of the EVs, we previously observed unspecific signals derived from non-EVs particles within the antibody’s solutions. To overcome the problem, besides staining EVs with PKH26 we designed the experiment including control samples for single antibodies only, EVs flow buffer, EVs flow buffer + PKH26, and unstained EVs, to remove all the unspecific signals.

### Transmission electron microscopy (TEM)

Isolated EVs were incubated on formvar/carbon-coated 100-mesh copper grids for 10 min, washed with water, and stained with 2% uranyl acetate aqueous solution for 1 min. Grids were viewed using a JEOL 1200EX II transmission electron microscope and photographed using a Gatan digital camera.

### Mouse HNSCC xenograft

All mice were housed, treated, and handled in accordance with the guidelines set forth by the University of California, San Diego Institutional Animal Care and Use Committee (IACUC), and the National Institutes of Health’s Guide for the Care and Use of Laboratory Animals. In all, 8–10 weeks female NOD/SCID/γc^−/−^ (NSG) mice (Jackson Laboratories) were sub-lethally irradiated (225 cGy) 1 day prior to tumor engraftment. Cal27-WT or KO cells were harvested and resuspended in DPBS. In all, 6 × 10^6^ cells were inoculated subcutaneously on the right flank, and mice were randomly assigned to experimental groups. Five mice were used per group. 1 × 10^7^ NK cells resuspended in DPBS were injected i.v. when the tumor volume was ~50 mm^3^ and NK cells were maintained with injection of hIL15 (1 μg/mouse) every day and hIL-2 (80,000 IU/mouse) every other day for 7 days. Tumor volume was monitored using a digital caliper every 2–3 days (see schematic in Fig. 7). Mice were killed when loss of ability to ambulate was observed or at the experiment endpoint with rapid and humane method of euthanasia. The maximum tumor size allowed by our IACUC of 1500 mm^3^ was not exceeded in these studies. For assessing NK cell infiltration in tumors, tumor cells were inoculated in four mice per group as described above. In all, 1 × 10^7^ NK cells resuspended in DPBS were injected i.v. when the tumor volume was ~50 mm^3^ and at day 7 post treatment mice were killed and tumors dissected. Tumors were mechanically dissociated and filtered through a 75 μm cell strainer to obtain a single-cell suspension. Cells were divided into two samples, one for isotype controls and one stained with anti-human CD45 and anti-human CD56 antibodies following the protocol illustrated in the paragraph FACS. Samples were normalized per volume analyzed by the flow cytometer and the number of NK cells calculated on the total volume of the sample. To set up the gating strategy, tumor samples without NK cell treatment were mixed with 2 × 10^4^ human NK cells, stained with anti-human CD45 and anti-human CD56 antibodies or isotype controls, and analyzed. The gate on CD45^+^/CD56^+^ cells was then used for counting human NK cells in Cal27-WT or CHMP2A-KO tumor samples.

### TCGA HNSCC and GBM data sets analysis

Gene expression data from HNSCC and GBM tumors were obtained from TCGA. The gene sets representing NK cells were generated by performing differential expression analysis on the gene expression dataset from Novershtern et al. 2011 (GEO dataset accession GSE24759) containing mRNA profiles for 38 distinct purified populations of human hematopoietic cells^52^, and then taking the top 20 genes overexpressed and top 20 genes underexpressed in NK cells, compared to all other cell types. The gene set of overexpressed genes was named “NK_20_up” and the underexpressed one was named “NK_20_down”. Single sample GSEA (ssGSEA) was performed on the bulk TCGA tumor expression data with both gene sets to assess the degree of up and down enrichment of each HNSCC and GBM TCGA sample^73,74^. A final NK_20 enrichment score was created by subtracting the NK_20_down ssGSEA score from the NK_20_up ssGSEA for each HNSCC TCGA sample. This NK_20 score was then correlated with the profile of CHMP2A mRNA expression in the HNSCC and GBM TCGA samples (Supplemental Fig. 7) using the mutual information between those two profiles (Information Coefficient Λ^75^,). Survival analysis was also performed on the HNSCC and GBM TCGA datasets by dividing patients into a CHMP2A “high” and “low” groups using the median expression of CHMP2A (Supplemental Fig. 3). Correlation of CHMP2A with overall survival in HNSCC and GBM TCGA samples was analyzed using the Kaplan-Meier method^76^ and statistical significance was assessed using the standard Mantel–Cox log‐rank test.

### Quantification and statistical analysis

Data are presented as the mean ± standard error of the mean. Differences between groups were evaluated using the one-way analysis of variance (ANOVA), two-way ANOVA, or Two-tailed *t* test (noted in figure legends). For the quantification of nanoparticles, data collected on a NanoSight LM10 (Malvern Panalytical) were analyzed with the software NTA v3.4 (Malvern Panalytical) and are presented as the mean of five replicates ± standard error. Statistical analysis is performed in the environment of GraphPad Prism 8. All tests were considered significant at *p* < 0.05.

### Reporting summary

Further information on research design is available in the Nature Research Reporting Summary linked to this article.

## Supplementary information

Supplementary Information

Reporting Summary

## Data Availability

The Crispr Screen sequencing data that support the findings of this study have been deposited in GEO with the accession code GSE175862. The RNA sequencing data that support the findings of this study have been deposited in GEO with the accession code GSE175863. The TCGA publicly available data used in this study are available in the TCGA-GBM database and in the TCGA-HNSC database The source data underlying Figures and Supplementary Figures including raw blot and TEM images are provided as Source data file and also downloadable at the following link: 10.6084/m9.figshare.19207998. All the other data supporting the findings of this study are available within the article and its Supplementary Information files. Source data are provided with this paper.

## Source data

Source Data
