# Supplementary material for: CHMP2A regulates tumor sensitivity to natural killer cell-mediated cytotoxicity
Source: Nat Commun. 2022 Apr 7;13:1899. doi: 10.1038/s41467-022-29469-0 (PMC8990014; doi:10.1038/s41467-022-29469-0)
Supplement: Supplementary file 1 — Supplementary Information [file 41467_2022_29469_MOESM1_ESM.pdf]

Supplemental Information for:

CHMP2A regulates tumor sensitivity to natural killer cell-mediated cytotoxicity.

Bernareggi et al.,

Supplemental Figure 1

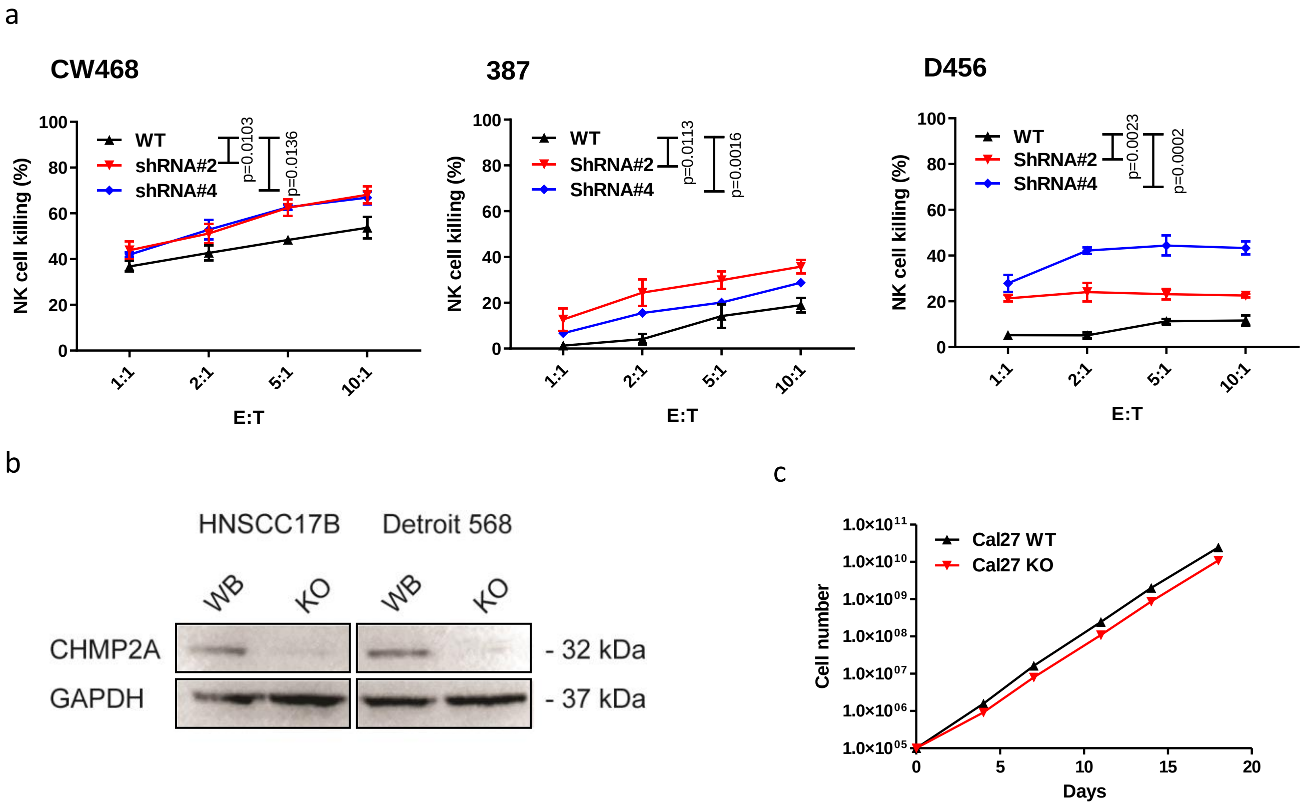

Supplemental Figure 1. **CHMP2A silencing increases GSC cell lines sensitivity to NK cells-mediated cytotoxicity**. a, 4-hours cytotoxicity assay to determine the effect of silencing CHMP2A in GSC-resistance to NK cell-mediated cytotoxicity. Two shRNAs were used (shRNA#2, shRNA#4). Error bars represent +/- SEM across n = 3 replicates. Statistical analysis was performed by one-tailed paired Student t-Test. Representative of n = 2 independent experiments. b, immunoblot analysis of WT and KO Detroit568 and HNSCC17B cell lines KO for CHMP2A. GAPDH serves as a loading control. c, Growth curve of Cal27 WT and CHMP2A KO cells shows no significant difference in cell expansion rate in 18 days.

Supplemental Figure 2

a

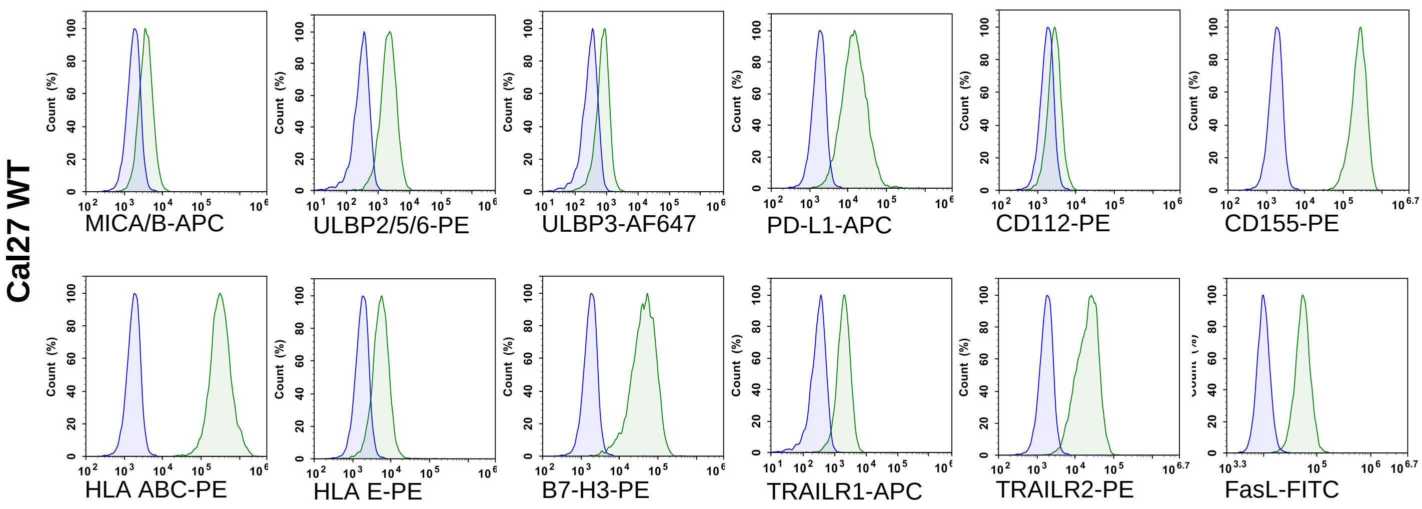

b

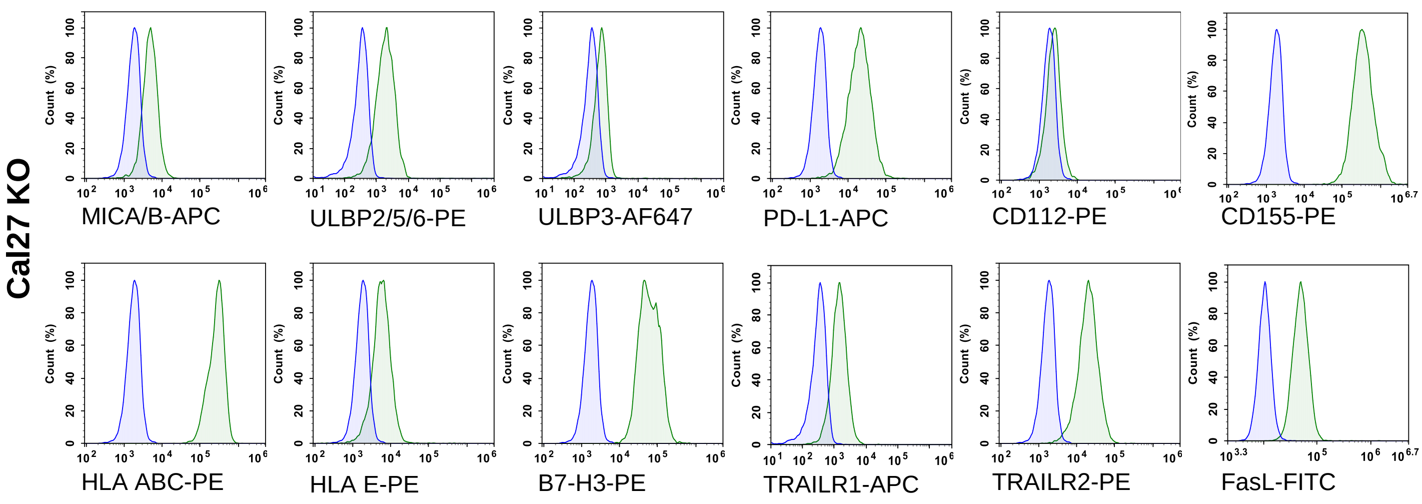

Supplemental Figure 2. CHMP2A KO and WT Cal27 cells NK cell ligands panel. a, Cal27 WT, and **b**, CHMP2A KO analyzed by flow cytometry for known NK cell activating or inhibitory ligands. Histograms show no significant differences in the mean fluorescence of MICA/B, ULBP2/5/6, ULPB3, PD-L1, CD112, CD155, HLA ABC, HLA E, B7-H3, TRAILR1, TRAILR2 and FasL (green histograms). In blue is shown the isotype control.

Supplemental Figure 3

a

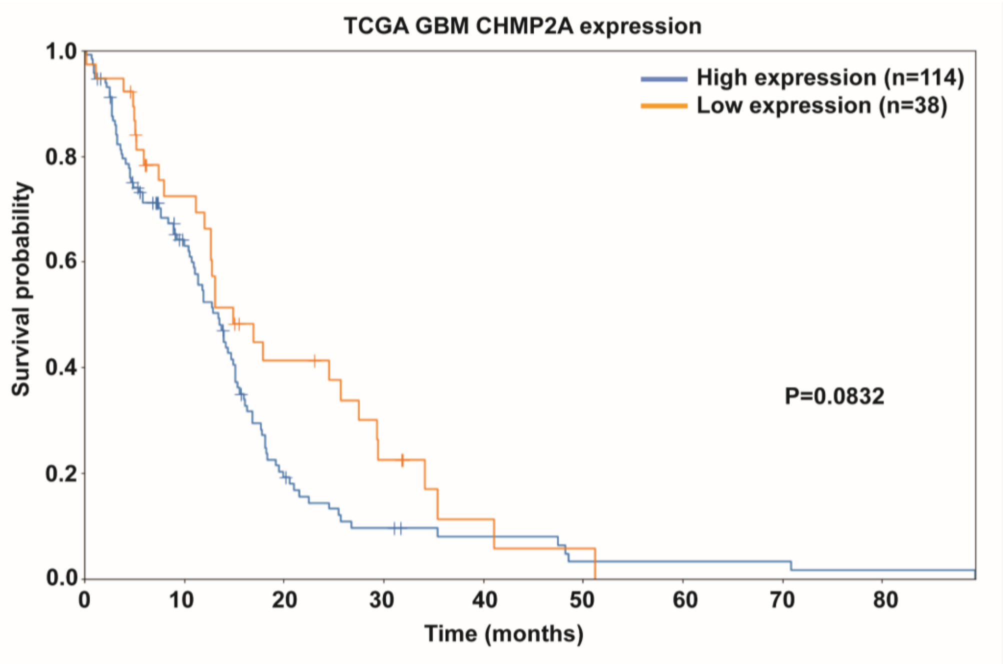

b

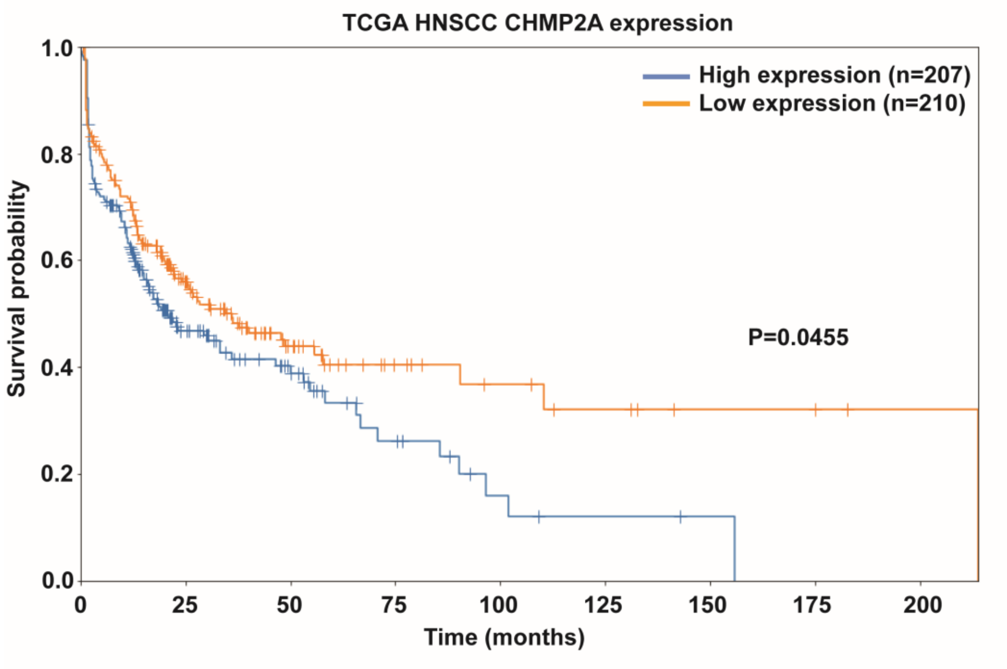

Supplemental Figure 3. TCGA data analysis for CHMP2A in GBM and HNSCC datasets. Tumor mRNA expression levels were obtained from TCGA for n=152 patients with GBM and n=417 patients with HNSCC. Kaplan-Maier curve showing survival probability in **a**, GBM and **b**, HNSCC based on CHMP2A expression levels. Statistical significance was assessed using the standard Mantel–Cox log-rank test.

Supplemental Figure 4

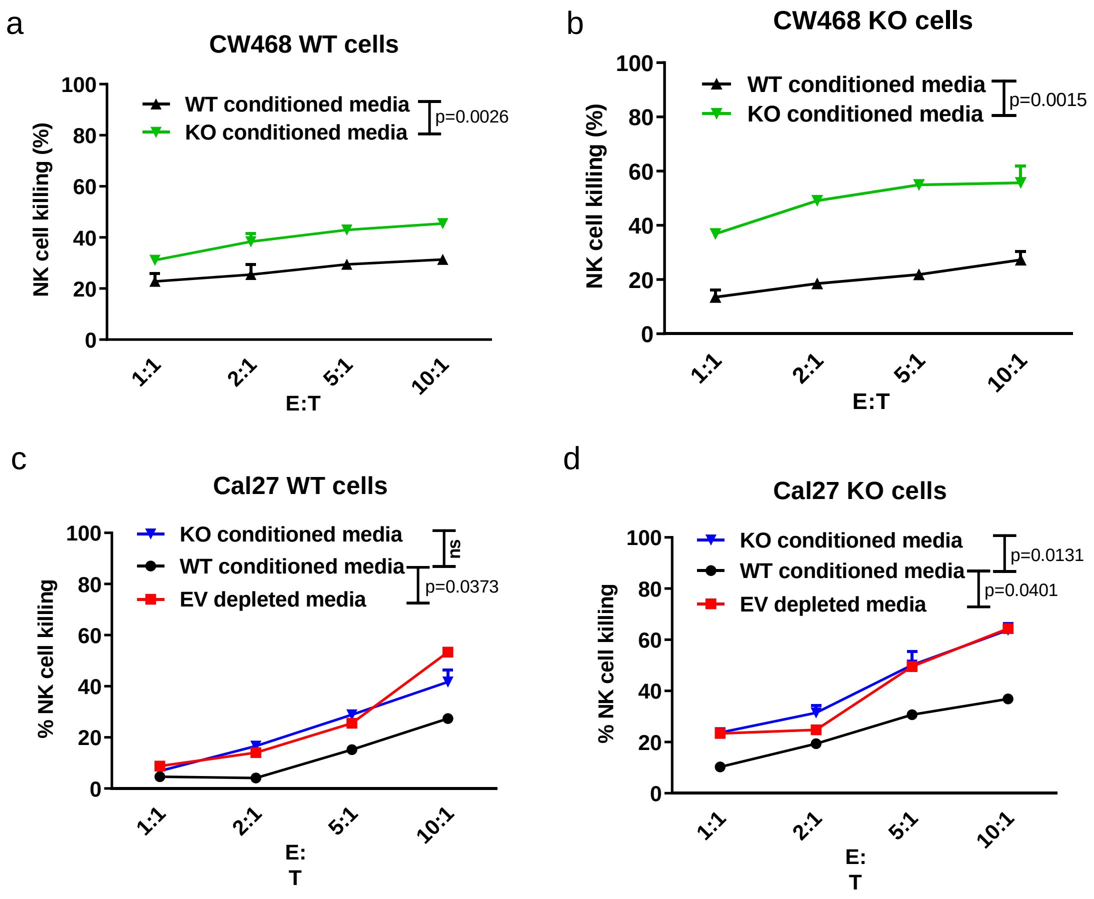

Supplemental Figure 4. CHMP2A KO conditioned media increases CW468 and Cal27 sensitivity to NK cells-mediated cytotoxicity. a, **b**, 4-hours cytotoxicity assay to determine the effect of CW468 WT and *CHMP2A* KO conditioned media on CW468 WT-resistance to NK cell-mediated cytotoxicity. **c**, **d**, 4-hours cytotoxicity assay to determine the effect of Cal27 WT and *CHMP2A* KO conditioned media on CW468 WT-resistance to NK cell-mediated cytotoxicity. EVs depleted media was also tested on Cal27 showing similar results as using *CHMP2A* KO conditioned media. The error bars represent standard error from the mean (+/- SEM) across n = 3 replicates. Statistical analysis was performed by one-tailed paired Student t-Test. Representative of n = 2 independent experiments.

Supplemental Figure 5

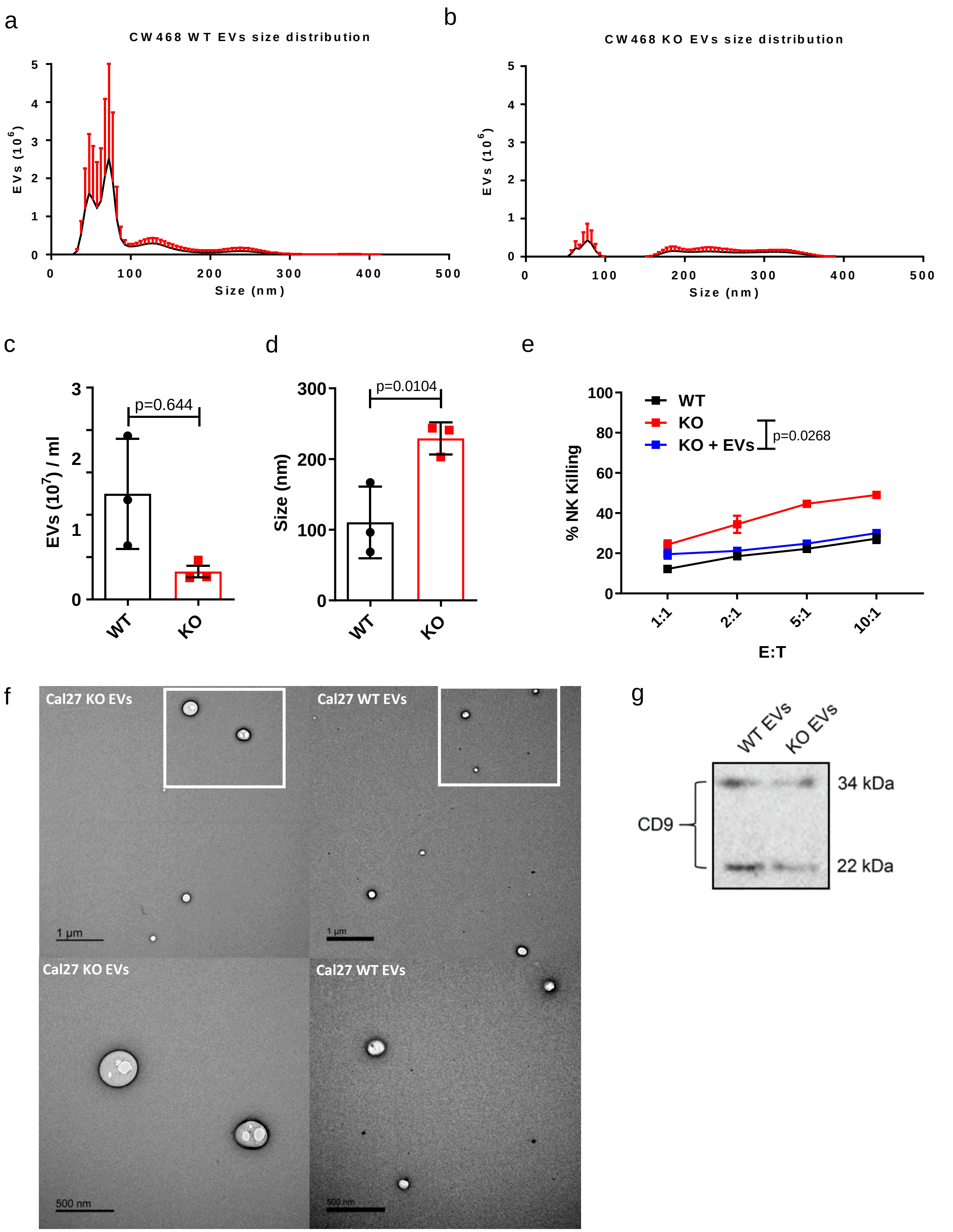

Supplemental Figure 5. CW468-derived EVs secretion is reduced in *CHMP2A*-KO cells. In **a, b**, charts showing the size distribution and number of EVs secreted from CW468 WT (**a**) and *CHMP2A* KO (**b**) cells. Samples were loaded on a Nanosight LM10 and analyzed for 1 minute for each of n = 3 technical replicates and the error bars represent +/- SEM across n = 3. **c**, comparison of EVs number in CW468 WT and *CHMP2A* KO determined on 10 μl of EVs suspension diluted in 1 ml of DPBS. **d**, average EVs size analyzed during nanoparticle tracking of CW468 WT and *CHMP2A* KO derived EVs. **e**, comparison of EVs number in Cal27 WT treated with tipifarnib (Tip) and the corresponding DMSO control (CTRL) calculated on 10 μl of EVs suspension diluted in 1 ml of DPBS. **c**-**d,** error bars represent +/- SEM across n = 3 technical replicates and unpaired two-tailed Student's t-test was performed to determine statistical significance. **e**, 4-hours cytotoxicity assay using NK cells as effectors against CW468 WT and *CHMP2A* KO. CW468 *CHMP2A* KO cells were treated with EVs from WT cells showing similar reduced sensitivity to NK cell killing as CW468 WT cells. Error bars represent +/- SEM, n = 3 replicates. Statistical analysis was performed by two-way ANOVA and Bonferroni's post hoc multiple comparison test. **f**, Representative TEM images from EVs secreted by *CHMP2A* KO (top row) and Cal27 WT (bottom row). Images shows sparse EVs with no aggregates and bigger EVs from *CHMP2A* KO cells. **g**, Immunoblot analysis of CD9 on Cal27 WT and *CHMP2A* KO EVs lysates. The same volume of EVs lysate was loaded, showing the diminished CD9 intensity correlates with lower number of EVs in KO cells. **a-d**, Representative of n = 3 independent experiments. **e-g**. Representative of n = 2 independent experiments.

Supplemental Figure 6

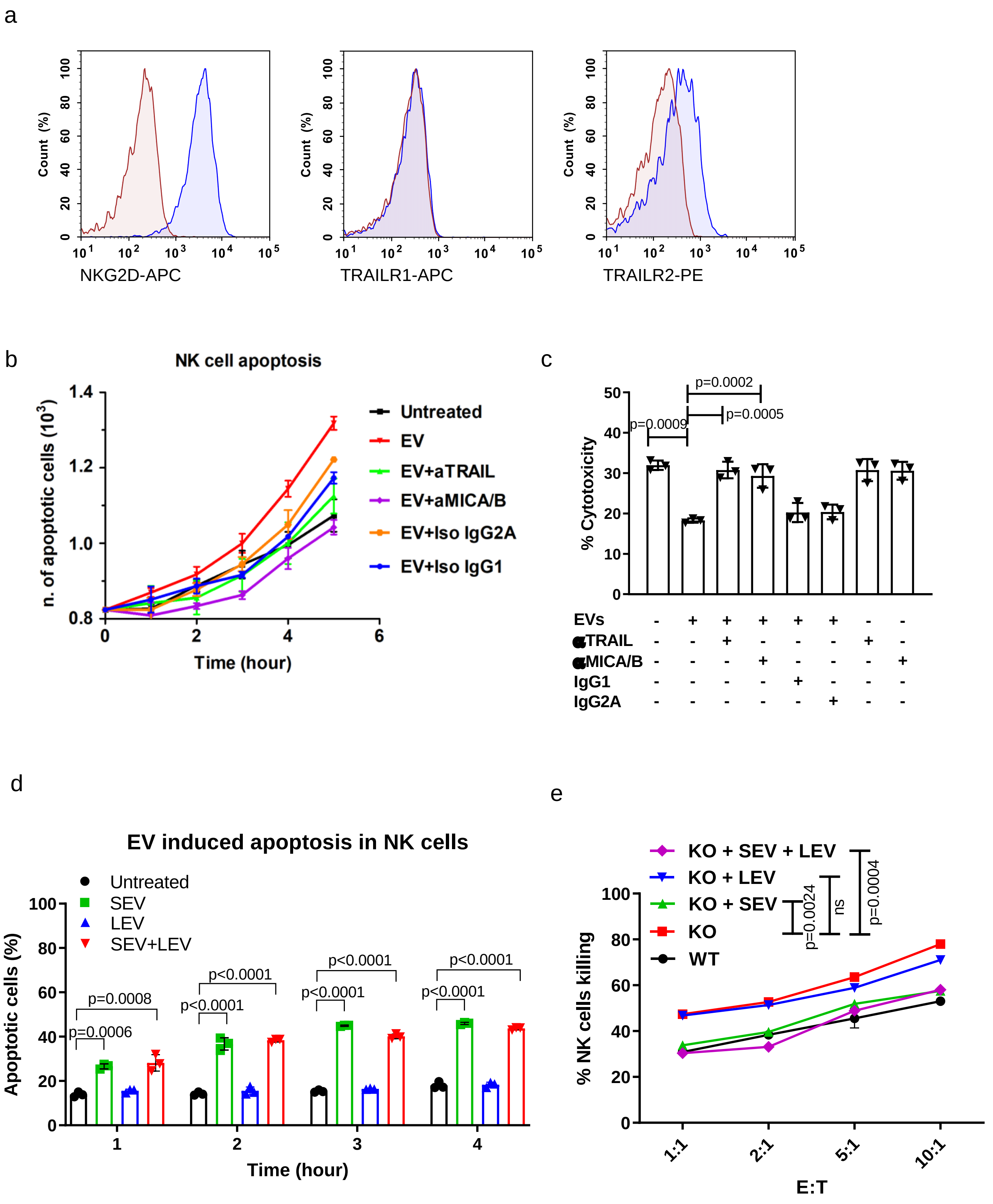

Supplemental Figure 6. NK cells express NKG2D and TRAILR2. **a**, Flow cytometry to measure the expression of NKG2D, TRAILR1 and TRAILR2 on NK cells. Blue histograms show NK cells express NKG2D and TRAILR2 while no difference with the isotype control (red histogram) is observed in the mean fluorescence of TRAILR1. **b**, enlargement of the first five hours of the NK cell apoptosis assay showed in Figure 6c. It is possible to appreciate comparable apoptosis levels as shown in Figure 6b. **c**, antibodies blocking MICA/B and TRAIL (aMICA/B and aTRAIL) limit the inhibitory effect of EV on NK cells-mediated killing. 4-hours cytotoxicity assay using NK cells as effectors against K562 cells (5:1). **d**, Flow cytometry CASP3/7 viability assay showing increasing NK cell death once exposed to SEV, LEV or a combination of the two from Cal27 in a time course of 4-hours. Error bars represent +/- SEM across n = 3 replicates, two-way ANOVA and Bonferroni's post hoc test was performed to determine statistical significance. **e**, 4-hours cytotoxicity assay using NK cells as effectors against Cal27 WT or KO as target cells. Here we show how Cal27 derived SEV reduce the killing in Cal27 KO while LEV did not. Error bars represent +/- SEM across n = 3 replicates. Statistical analysis was performed by one-way ANOVA and Bonferroni's post hoc test (ns, not significant). **a-e**, Representative of n = 2 independent experiments.

Supplemental Figure 7

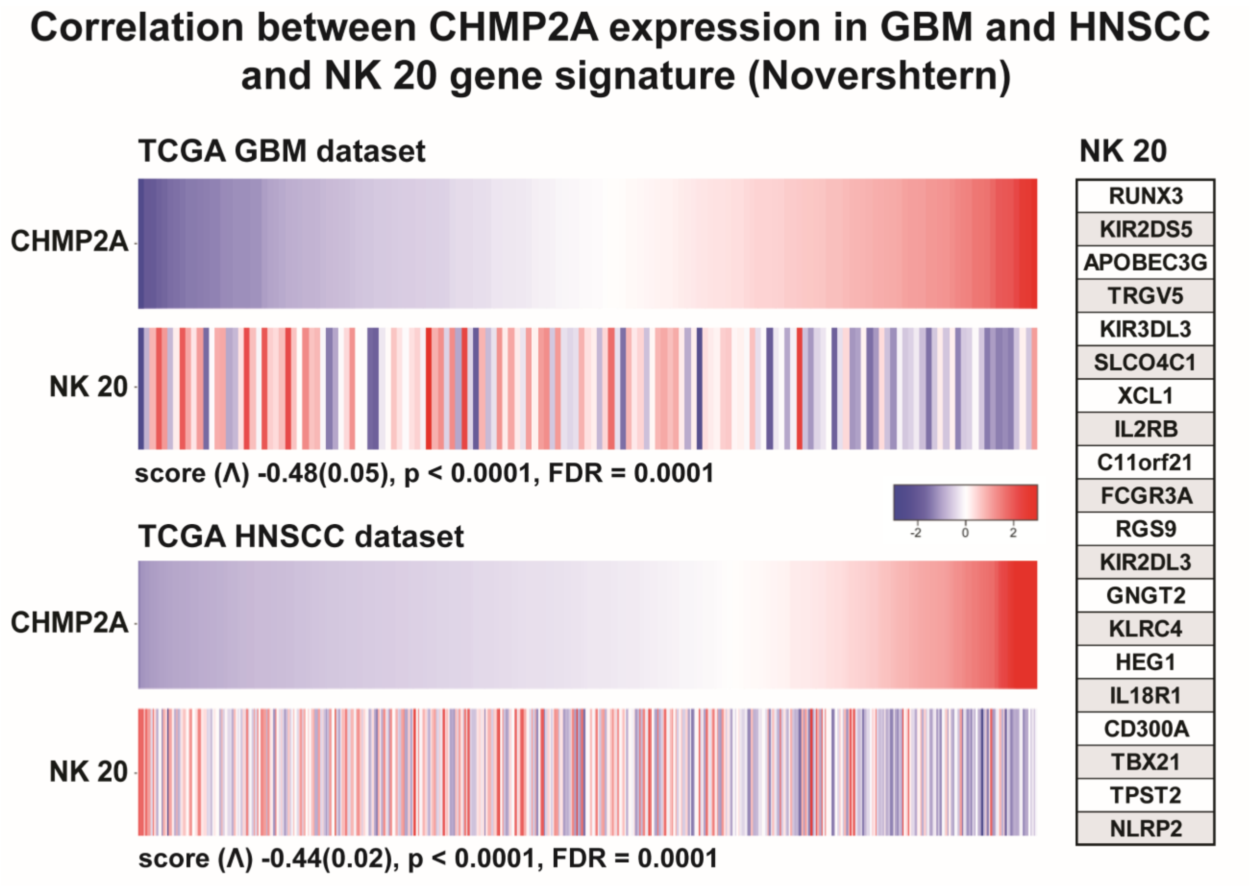

Supplemental Figure 7. Heatmap showing the correlation between low CHMP2A expression and NK cells signature on GBM and HNSCC gene expression data sets. The gene sets representing NK cells were generated by performing differential expression analysis on the gene expression dataset from Novershtern et al. 2011 [1]. The score was correlated with the profile of CHMP2A mRNA expression in the HNSCC and GBM TCGA samples using the mutual information between those two profiles (Information Coefficient[2]). TCGA-GBM dataset n=152, TCGS-HNSC dataset n=417.

References:

1 - Novershtern, N. et al. Densely interconnected transcriptional circuits control cell states in human hematopoiesis. Cell 144, 296-309, doi:10.1016/j.cell.2011.01.004 (2011).

2 - Kim, J. W. et al. Characterizing genomic alterations in cancer by complementary functional associations. Nature biotechnology 34, 539-546, doi:10.1038/nbt.3527 (2016).
