# Supplementary material for: CHMP2A regulates tumor sensitivity to natural killer cell-mediated cytotoxicity
Source: Nat Commun. 2022 Apr 7;13:1899. doi: 10.1038/s41467-022-29469-0 (PMC8990014; doi:10.1038/s41467-022-29469-0)
Supplement: Supplementary file 2 — Reporting Summary [file 41467_2022_29469_MOESM2_ESM.pdf]

nature research

Corresponding author(s): Dan S. Kaufman

Last updated by author(s): Feb 21, 2022

Reporting Summary

Nature Research wishes to improve the reproducibility of the work that we publish. This form provides structure for consistency and transparency in reporting. For further information on Nature Research policies, see our Editorial Policies and the Editorial Policy Checklist.

Statistics

For all statistical analyses, confirm that the following items are present in the figure legend, table legend, main text, or Methods section.

n/a	Confirmed	
☐	☒	The exact sample size (*n*) for each experimental group/condition, given as a discrete number and unit of measurement
☐	☒	A statement on whether measurements were taken from distinct samples or whether the same sample was measured repeatedly
☐	☒	The statistical test(s) used AND whether they are one- or two-sided<br>*Only common tests should be described solely by name; describe more complex techniques in the Methods section.*
☐	☒	A description of all covariates tested
☐	☒	A description of any assumptions or corrections, such as tests of normality and adjustment for multiple comparisons
☐	☒	A full description of the statistical parameters including central tendency (e.g. means) or other basic estimates (e.g. regression coefficient) AND variation (e.g. standard deviation) or associated estimates of uncertainty (e.g. confidence intervals)
☐	☒	For null hypothesis testing, the test statistic (e.g. *F*, *t*, *r*) with confidence intervals, effect sizes, degrees of freedom and *P* value noted<br>*Give P values as exact values whenever suitable.*
☒	☐	For Bayesian analysis, information on the choice of priors and Markov chain Monte Carlo settings
☒	☐	For hierarchical and complex designs, identification of the appropriate level for tests and full reporting of outcomes
☐	☒	Estimates of effect sizes (e.g. Cohen's *d*, Pearson's *r*), indicating how they were calculated

Our web collection on statistics for biologists contains articles on many of the points above.

Software and code

Policy information about availability of computer code

Data collection	NovoExpress V3.4 (serial: 451180228922), Nanosight NTA3.1 (C11440-63)
Data analysis	NovoExpress V3.4 (serial: 451180228922), Nanosight NTA3.1 (C11440-63)

For manuscripts utilizing custom algorithms or software that are central to the research but not yet described in published literature, software must be made available to editors and reviewers. We strongly encourage code deposition in a community repository (e.g. GitHub). See the Nature Research guidelines for submitting code & software for further information.

Data

Policy information about availability of data

All manuscripts must include a data availability statement. This statement should provide the following information, where applicable:

- Accession codes, unique identifiers, or web links for publicly available datasets
- A list of figures that have associated raw data
- A description of any restrictions on data availability

All data generated or analyzed during this study are included in this published article (and its supplementary information files). Accession codes from data generated have been provided in the manuscript text

Field-specific reporting

Life sciences study design

All studies must disclose on these points even when the disclosure is negative.

Sample size	Sample sizes were chosen based on preliminary data of tumor growth in this mouse model. Sample size was determined to be adequate based on the consistency of measurable differences within and between groups. The number of mice is rationalized following the “resource equation method” described in Festing et al., 2002 (Festing MF, Altman DG. Guidelines for the design and statistical analysis of experiments using laboratory animals. ILAR journal. 2002;43(4):244-58).
Data exclusions	No data have been excluded from the analysis
Replication	Every experiment was replicated at least three with near-identical results.
Randomization	Based on the tumor volumes on the first day of treatment, tumor bearing mice were randomly assigned to treatment groups such that each treatment group had the same average tumor volume of 50 mm cube. Experiments other than mice experiments were not randomized because cells were collected from the same flask and no variation in cell growth or phenotype between samples is possible.
Blinding	The data presented did not require the use of blinding. Data reported for mouse experiments were not subjective but rather based on quantitative analyses.

Reporting for specific materials, systems and methods

We require information from authors about some types of materials, experimental systems and methods used in many studies. Here, indicate whether each material, system or method listed is relevant to your study. If you are not sure if a list item applies to your research, read the appropriate section before selecting a response.

Materials & experimental systems

n/a	Involved in the study	
☐	☒	Antibodies
☐	☒	Eukaryotic cell lines
☒	☐	Palaeontology and archaeology
☐	☒	Animals and other organisms
☒	☐	Human research participants
☒	☐	Clinical data
☒	☐	Dual use research of concern

Methods

n/a	Involved in the study	
☒	☐	ChIP-seq
☐	☒	Flow cytometry
☒	☐	MRI-based neuroimaging

Antibodies

Antibodies used

Flow cytometry.
PE-Cy7 anti-human CD56 mlgG1#, BioLegend, clone HCD56, cat. n. 318318, lot. n. B336511
APC anti-human CD45, mlgG1#, BD Biosciences, clone H130, cat. n. 555485, lot. n.9291059
APC anti-human MICA/MICB Antibody, Biolegend, clone 6D4, cat. n. 320907, lot.n. B263218
Human TRAIL R2/TNFRSF10B PE-conjugated Antibody - R&D, cat. n. FAB6311P
PE anti-human ULBP-2/5/6, R&D, clone 165903, cat. n. FAB1298P, lot.n. LWE0718071
Human ULBP-3 Alexa Fluor® 647-conjugated Antibody, R&D, cat. n. FAB1517R, lot.n.1509994
PE anti-hCD178 (FasL), eBioscience, clone NOK-1, cat. n. 12-9919-42, lot.n.E11032-251
PE Mouse Anti-Human CD112, BD Biosciences, cat. n. 551057, lot.n.8102515
PE Mouse Anti-Human HLA-ABC, BD Biosciences, cloneSKIL4, cat. n. 557349, lot.n. B268062
PE anti-CD155 antibody, Biolegend, clone 3D12, cat. n. 337609, lot.n.B200041
PE anti-HLA E antibody, Biolegend, cat. n. 342603
APC anti-human CD274 (B7-H1, PD-L1) Antibody, Biolegend, clone 29E2A3, cat. n. 329707, lot.n. 13292681
PE anti-human CD276 (B7-H3) Antibody [Clone: MIH42], Biolegend, cat. n. 351003, lot.n.B186625
PE anti-human CD262 (DR5, TRAIL-R2) Antibody [Clone: DJR2-4 (7-8)], Biolegend, cat. n. 307405
APC anti-human CD261 (DR4, TRAIL-R1) Antibody [Clone: DJR1], Biolegend, cat. n. 307207
APC Mouse Anti-Human CD314 (NKG2D), BD Biosciences, cat. n. 558071., lot.n.9199502

Functional assays.
Purified anti-human MICA/MICB [Clone: 6D4], Biolegend, cat. n. 320902, lot.n.B263218
Human TRAIL/TNFSF10 Antibody, R&D, clone 75411, cat. n. MAB375, lot.n.COX042006A
Purified Mouse IgG2a, # Isotype Ctrl Antibody, Biolegend, clone MG2a-53, cat. n. 401501, lot.n. B243565
Purified Mouse IgG1, # Isotype Ctrl Antibody, Biolegend,clone MG1-45, cat. n. 401401, lot.n. B306245
Human BD Fc Block, BD Pharmingen, cat n. 564220, lot.n.9137784

ImmunoBlot.

	CHMP2A Antibody, rabbit policlonal, Proteintech, cat. n. 10477-1-AP, lot.n.00046622<br>GAPDH Loading Control Monoclonal Antibody (GA1R), Invitrogen, cat. n. MA5-15738, lot.n.WC318870<br>CD9, clone (D8O1A) Rabbit mAb, Cell Signaling, cat. n. 13174S
Validation	R&D: It is hereby certified that the above product has been tested for proper performance and function under our established Quality Control testing criteria.<br>Biolegend: This product lot has passed BioLegend's QC testing and is certified for use. For details on QC testing view our page at biolegend.com/en-us/quality-control.<br>BD Biosciences: This Product Complies with all BDB release criteria.BD Biosciences, Life Science Research Reagents, is a registered facility with the British Standards Institute (BSI) for ISO 9001:2008. It is authorized by our Quality Assurance program to be released for sale

Eukaryotic cell lines

Policy information about cell lines

Cell line source(s)	Glioblastoma stem cell lines 387, 1517, CW468 and D456 were provided by Jeremy Rich. Head and neck squamous cells carinoma cell lines Cal27, Detroit568 and HNSCC17B were provided by J. Silvio Gutkind. K562 were purchased from ATCC
Authentication	None of these cell lines has been authenticated in our lab
Mycoplasma contamination	All the cell lines were frequently tested for mycoplasma contamination. No presence of mycoplasma contamination was detected according to PCR test.
Commonly misidentified lines<br>(See ICLAC register)	No commonly misidentified cells were used in this study

Animals and other organisms

Policy information about studies involving animals; ARRIVE guidelines recommended for reporting animal research

Laboratory animals	In the present study 8-10 weeks female NOD/SCID/γc-/- (NSG) mice (Jackson Laboratories) have been used. Housing conditions: Temperature 71°F, humidity (high/low %) 48/21, dark/light cycle 6pm-6am
Wild animals	No wild animals have been used in this study
Field-collected samples	No field-collected samples have been used in this study
Ethics oversight	All mice were housed, treated, and handled in accordance with the guidelines set forth by the University of California, San Diego Institutional Animal Care and Use Committee and the National Institutes of Health's Guide for the Care and Use of Laboratory Animals.

Note that full information on the approval of the study protocol must also be provided in the manuscript.

Flow Cytometry

Plots

Confirm that:

- [ ] The axis labels state the marker and fluorochrome used (e.g. CD4-FITC).
- [x] The axis scales are clearly visible. Include numbers along axes only for bottom left plot of group (a 'group' is an analysis of identical markers).
- [x] All plots are contour plots with outliers or pseudocolor plots.
- [x] A numerical value for number of cells or percentage (with statistics) is provided.

Methodology

Sample preparation	Cell lines were trypsinized and centrifuged at 1500 rpm for 5 min while NK cells resuspended in media and centrifuged at 1500 rpm for 5 minutes. Cells were resuspended in DPBS + 2% FCS (flow buffer) and stained with trypan blue, counted using an inverted microscope and 1 x 10^5 cells were dispensed per sample. Cells were incubated on ice in the dark for 20 minutes in flow buffer containing the antibodies of interest. Stained cells were centrifuged at 1500 rpm for 5 min and washed flow buffer two times. Finally, cells were resuspended in 300 µl of flow buffer containing SYTOX Blue Dead Cell Stain (Life Technologies) diluted by a factor of 1,000 and analyzed by FACS.
Instrument	ACEA Bioscience Inc. NovoCyte 3000
Software	NovoExpress V3.4

Cell population abundance	Cell were not sorted in this study.
Gating strategy	Cells were gated on the FSC/SSC plot. The gated cells were sequentiality gated to remove doublets on a SSC-H/SSC-H plot and live cells were than gated on AmCyan channel. The live cells were analyzed for the markers of interest and showed on histograms. A supplemental file showing the gating strategies has been provided as supplemental information.

☒ Tick this box to confirm that a figure exemplifying the gating strategy is provided in the Supplementary Information.
